# Ketamine for the treatment of mental health and substance use disorders: comprehensive systematic review

**DOI:** 10.1192/bjo.2021.1061

**Published:** 2021-12-23

**Authors:** Zach Walsh, Ozden Merve Mollaahmetoglu, Joseph Rootman, Shannon Golsof, Johanna Keeler, Beth Marsh, David J. Nutt, Celia J. A. Morgan

**Affiliations:** Department of Psychology, University of British Columbia, Canada; Psychopharmacology and Addiction Research Centre, Department of Psychology, University of Exeter, UK; Department of Psychology, University of British Columbia, Canada; Department of Psychology, University of British Columbia, Canada; Eating Disorders Research Group, Department of Psychological Medicine, Institute of Psychiatry, Psychology & Neuroscience, Kings College London, UK; Psychopharmacology and Addiction Research Centre, Department of Psychology, University of Exeter, UK; and Clinical Psychopharmacology Unit, Department of Clinical, Educational and Health Psychology, University College London, UK; Drug Science, UK; and Neuropsychopharmacology Unit, Division of Psychiatry, Department of Brain Sciences, Imperial College London, UK; Psychopharmacology and Addiction Research Centre, Department of Psychology, University of Exeter, UK

**Keywords:** Alcohol disorders, anxiety disorders, depressive disorders, mental health disorders, ketamine

## Abstract

**Background:**

In the past two decades, subanaesthetic doses of ketamine have been demonstrated to have rapid and sustained antidepressant effects, and accumulating research has demonstrated ketamine's therapeutic effects for a range of psychiatric conditions.

**Aims:**

In light of these findings surrounding ketamine's psychotherapeutic potential, we systematically review the extant evidence on ketamine's effects in treating mental health disorders.

**Method:**

The systematic review protocol was registered in PROSPERO (identifier CRD42019130636). Human studies investigating the therapeutic effects of ketamine in the treatment of mental health disorders were included. Because of the extensive research in depression, bipolar disorder and suicidal ideation, only systematic reviews and meta-analyses were included. We searched Medline and PsycINFO on 21 October 2020. Risk-of-bias analysis was assessed with the Cochrane Risk of Bias tools and A Measurement Tool to Assess Systematic Reviews (AMSTAR) Checklist.

**Results:**

We included 83 published reports in the final review: 33 systematic reviews, 29 randomised controlled trials, two randomised trials without placebo, three non-randomised trials with controls, six open-label trials and ten retrospective reviews. The results were presented via narrative synthesis.

**Conclusions:**

Systematic reviews and meta-analyses provide support for robust, rapid and transient antidepressant and anti-suicidal effects of ketamine. Evidence for other indications is less robust, but suggests similarly positive and short-lived effects. The conclusions should be interpreted with caution because of the high risk of bias of included studies. Optimal dosing, modes of administration and the most effective forms of adjunctive psychotherapeutic support should be examined further.

## Background

Ketamine is an N-methyl-d-Aspartate receptor agonist with well-established safety and efficacy as an analgesic and anaesthetic. Since ketamine was developed in 1964, largely as a replacement for phencyclidine, it has been used primarily in veterinary and paediatric anaesthesia, but interest in recent years has burgeoned in psychiatry after reports of its rapid-acting antidepressant effects.^1^ The potential for ketamine to be used in the treatment of psychiatric disorders was first noted in the 1970s,^2^ and has been the focus of formal investigation since the 1990s, with investigative and off-label use in the context of mental health increasing across North America and Europe since that time. The first approval by the USA Food and Drug Administration (FDA) of a ketamine-derived therapy for mental health came in 2019 for intranasal esketamine (the S-enantiomer of ketamine) as augmentation therapy for treatment-resistant depression, which has increased clinical and public interest internationally.

The past two decades have seen the development of ketamine for treatment of a broad range of mental health conditions beyond depression. Although a number of meta-analyses and systematic reviews have evaluated the use of ketamine in depression,^3,4^ there is less synthesised literature across other psychiatric uses. The current review integrates parallel reviews of the nascent research on ketamine as a treatment for mental health conditions and related research germane to the therapeutic potential of ketamine for mental health more broadly. This work is timely as off-label prescribing of ketamine for a large number of psychiatric disorders other than depression continues,^5^ but there have been no comprehensive syntheses of the state of the evidence so far.

## Route of administration, dose and therapeutic support

Route of administration is a key consideration in the use of ketamine for disorders such as depression, in which repeated or semi-regular dosing may be indicated.^3^ Intravenous is most widely used route of administration in clinical settings because of its superior bioavailability and control of dose. However, other modes of administration, such as oral, intramuscular, sublingual and intranasal, which require less clinical resources, have clear practical advantages for repeated dosing and patient comfort.^3,6–9^

Psychoactive and therapeutic effects of ketamine have also been reported to vary substantially by dose and route of administration.^10,11^ For example, the ketamine-assisted psychotherapy (KAP) model proposes distinct applications according to dose and mode, with lower-dose sublingual administration recommended for sessions that involve more active therapist–patient communication, and higher-dose intramuscular administration recommended for sessions that adhere more closely to current models of psychedelic psychotherapy with an inward focus, eye coverings and music.^12^ However, studies that have compared modes of administration have generally not reported meaningful differences in efficacy.^13,14^

Research on the ketamine dose–response relationship in the context of mental health is equivocal.^15^ Early studies to treat addiction used higher doses of 2–3.0 mg/kg administered via intramuscular injection,^16–19^ whereas more recent studies typically use 0.4 mg/kg or 0.5 mg/kg administered intravenously, infused over 40–60 min.^20–22^ Ketamine effects in depression generally last from a few days to 2 weeks,^15^ so repeated dosing is usually necessary to extend recovery. For example, the FDA-approved dosing model for esketamine includes an induction phase of twice weekly dosing, tapering down to a maintenance phase of weekly and later biweekly dosing, with no maximum period indicated.^23^ In general, further empirical work is required to confidently assert an optimal model of ketamine administration.

Psychoactive drugs have been proposed to work synergistically with psychological therapies,^24^ and support for preparation and integration are recognised as essential components of psychedelic-assisted psychotherapy.^25^ However, distinct psychotherapeutic support has not typically been emphasised in current psychiatric use of ketamine. The FDA-approved model of esketamine for treatment-resistant depression does not explicitly demand therapist engagement in preparation or integration of experiences. The relative lack of emphasis on adjunctive psychotherapy contrasts with the early work in ketamine-assisted psychotherapy by Salvador Roquet and his colleagues in Mexico in the 1970s^26^^,^^27^ which was embedded in a psychodynamically informed group therapy. Subsequent work in addiction also administered ketamine in the context of psychodynamic group therapy,^17^ and follow-up assessments of these groups suggested this intensive therapy programme was key to the effectiveness of ketamine in the treatment of alcohol use disorder.^28^ More recently, adjunct cognitive–behavioural therapy following ketamine infusions was found to prolong the therapeutic effects of ketamine for depression.^29^ The increasingly prominent KAP model also strongly emphasises the role of both therapeutic support and set and setting in accentuating and extending the longevity of ketamine effects.^12^

Approaches to ketamine therapy that involve active therapeutic support, such as KAP, leverage the subjective psychoactive effects of ketamine administration to maximise therapeutic benefit.^12^ Indeed, although the role of the acute psychoactive effects of ketamine remains a subject of debate, theory and research suggests that these effects may be important, and perhaps even central. For example, a ketamine-assisted psychotherapy session using a single psychedelic dose of ketamine produced higher rates of abstinence from heroin users than did a low sub-psychedelic dose.^18^ Further, an analysis of data from several studies of in-patient use of ketamine for the treatment of depression found that dissociative effects during the infusion were correlated with the antidepressant response.^30^ Another study in cocaine users identified a strong mediating effect of mystical experiences on ketamine-related reductions in cocaine use and craving, leading the authors to call for approaches to ketamine therapy that explicitly evoke and capitalise on the salutary effects of mystical experiences.^31^ However, a broad systematic review of the associations between psychoactive effects and ketamine treatment outcomes was inconclusive.^32^

## The current review

Although a number of meta-analyses and systematic reviews have evaluated the use of ketamine in depression,^3,4^ little work thus far has synthesised literature across other psychiatric uses of ketamine. The current review aims to evaluate the therapeutic benefits of ketamine treatment on psychiatric disorders, including depression, bipolar disorder, social and generalised anxiety, obsessive–compulsive disorders, post-traumatic stress disorders, substance use disorders and eating disorders. The review will also assess whether ketamine anaesthesia for electroconvulsive therapy (ECT) is effective for the treatment of these psychiatric disorders, and any adverse effects associated with ketamine treatment.

## Method

Methods of the systematic review were specified in advance and documented in a protocol registered on PROSPERO (identifier: CRD42019130636). The systematic review was conducted and reported in line with the Preferred Reporting Items for Systematic Reviews and Meta-Analyses (PRISMA) 2020 guidelines^33^.

### Eligibility criteria

We included any human studies investigating the clinical effect of ketamine, S(+)-ketamine (esketamine) or R(−)-ketamine (arketamine) in the treatment of mental health disorders. All peer-reviewed and published reports in English were eligible except for case studies, letters to editors and comments. Because of the voluminous research on ketamine's therapeutic effects in major depressive disorder (MDD), bipolar disorder and suicidal ideation, we limited our review for these topics to systematic reviews and meta-analyses. We excluded studies with a primary focus on (a) modelling schizophrenia and psychosis symptoms, (b) solely the mechanisms of action or predictors of treatment response, (c) ketamine metabolites only (i.e. hydroxyketamine, norketamine, dehydronorketamine and hydroxynorketamine), (d) the use of ketamine for treatment of general health conditions, (e) the use of ketamine as an analgesic or anaesthetic agent and (f) interventions for the treatment of problematic ketamine use.

### Search strategy and information sources

Literature search strategies were developed by using medical subject headings (MeSH) and text words related to ketamine and mental health disorders (see Supplementary Materials available at https://doi.org/10.1192/bjo.2021.1061 for the full search strategy). We searched Medline (Ovid Interface, 1946 onward) and PsycINFO (Ovid Interface, 1806 onward). All searches were completed on 21 October 2020. The searches were limited to English language and human study participants, and no date limits were imposed on the search. To supplement identification of relevant articles, we scanned reference lists of included studies or relevant reviews identified through the search. Before the start of the systematic review, we also searched PROSPERO for any ongoing or recently completed systematic reviews in the field.

### Study selection

Four independent reviewers screened the titles and abstracts against the inclusion and exclusion criteria. Before independent screening, the reviewers screened titles and abstracts of 100 papers each to calculate the interrater reliability. The intraclass coefficient was 0.98. During the full-text screening stage, reviewers were trained in using study eligibility forms to ensure consistency. Discrepant ratings for inclusion criteria were resolved by consensus.

### Data extraction

Four independent reviewers extracted data from included reports, using data extraction forms that were piloted before use; this involved a separate form for studies reporting on primary data and another form for systematic reviews and meta-analyses. For papers reporting data from individual studies, we extracted the following information: trial design and setting, patient characteristics (demographics, mental health diagnosis, comorbidities), ketamine treatment (dosage, frequency, duration), any adjunctive treatment (psychological or pharmacological), type of control used (placebo, active comparisons), treatment outcomes (acute and follow-up) and adverse effects. For systematic reviews and meta-analyses, we extracted eligibility criteria, characteristics of studies included, risk-of-bias analysis of included studies, summary measures, synthesis of results, narrative summary and adverse reactions. See Supplementary material for both of the data extraction forms. We contacted one author (Professor P. Glue) regarding the overlap of samples in three of their papers, to avoid double counting.^34–36^ With Professor Glue's guidance, we dealt with the overlap of the samples by stating in our results which of the studies included the same participants.

### Synthesis methods

Results of individual studies included in the synthesis were presented in a separate table for each psychiatric diagnosis. Meta-analysis was not deemed appropriate because of the heterogeneity of dosage, number and methods of ketamine administrations, patient populations, study designs and outcome measures. The results were grouped according to patient population (MDD, bipolar disorder, suicidal ideation, generalised and social anxiety disorders, post-traumatic stress disorder, obsessive–compulsive disorders, substance use disorders and eating disorders) and by setting (ketamine in combination with ECT). Additionally, adverse effects across all included studies were synthesised together.

### Risk of bias

We used version 2 of the Cochrane Risk-of-Bias tool to rate the quality of randomised controlled trials (RCTs).^37^ For non-randomised studies, we used the Risk of Bias in Non-randomised Studies-of Interventions.^38^ The quality of systematic reviews and meta-analyses were assessed with the A Measurement Tool to Assess Systematic Reviews 2 (AMSTAR 2) Checklist.^39^ Four reviewers (O.M.M., J. R., S. G. and J.K.) were trained in the use of tools, and risk-of-bias judgements were made independently, with disagreements resolved among reviewers.

## Results

**Fig. 1:**
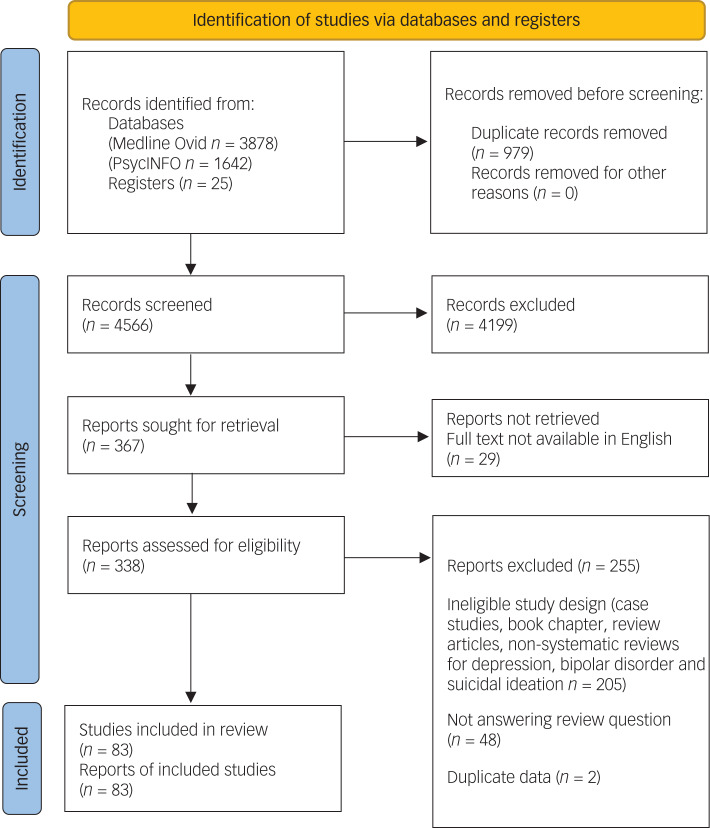
Preferred Reporting Items for Systematic Reviews and Meta-Analyses (PRISMA) 2020 flow diagram for new systematic reviews that included searches of databases and registers only. Diagram template obtained from Page MJ, McKenzie JE, Bossuyt PM, Boutron I, Hoffmann TC, Mulrow CD, et al. The PRISMA 2020 statement: an updated guideline for reporting systematic reviews. *BMJ* 2021; 372: n71.

We included 83 published reports in this systematic review (see Fig. 1 for PRISMA flow diagram), comprising 33 systematic reviews (of which 17 included meta-analyses), 29 RCTs, two randomised trials with no placebo control, three non-randomised trials with controls, six open-label trials and ten retrospective reviews. A list of studies that appeared to meet the inclusion criteria but were excluded, and reasons for exclusions, are provided in Supplementary Table 1.

### Unipolar depression and MDD

We identified 24 systematic reviews of the antidepressant effect of ketamine in the treatment of unipolar depression and/or MDD, 12 of which conducted meta-analyses. In general, reviews consistently identified a robust, rapid, and typically short-lived antidepressant effect for ketamine.

All systematic review or meta-analytic articles noted rapid onset of ketamine's antidepressant effect among patients with MDD.^3,4,6–9,14,15,40–53^ Meta-analyses, which primarily focused on a single intravenous infusions, identified lower mean depression severity and remission in ketamine groups relative to placebo, with onset between 1 and 24 h post-infusion,^6,9,14,40,41,43,45,47,50,52^ and typically persisting for 1–2 weeks.^3,8,9,14,41–43,45,47,49,50^ Repeated administrations of ketamine of up to six doses produce a more pronounced antidepressant effect at the 2-week follow-up relative to a single administration,^43,44,50^ and prolonged time to relapse.^3,15,44,47,50^

Studies on routes of administration other than intravenous signal an antidepressant effect comparable with that of intravenous administration.^4,9,14,44,46,52^ One meta-analysis demonstrated that adjunctive intranasal ketamine was significantly more effective than placebo for reducing depressive symptoms, response and remission from depression.^53^ A direct comparison of intravenous versus intramuscular ketamine for depression concluded that the methods were equivalent in terms of safety and effectiveness.^13^ With regards to oral ketamine, a systematic review reported that repeated administration of oral ketamine was well-tolerated and had medium-to-large effect sizes, with a delay of 2–6 weeks before the emergence of clinically significant antidepressant effects.^54^ A meta-analysis comparing intravenous, intranasal and oral ketamine administrations was inconclusive because of heterogeneity across studies, but noted that effects for intravenous ketamine appeared to peak at 2–6 days, compared with 24 h for intranasal administration and 7–20 days for oral administration, potentially because of increasing bioavailability over time.^55^

Ketamine is effective both in trials restricted to patients with treatment-resistant depression (i.e. non-response to prior antidepressant trials) and trials that included patients who had responded to prior treatment.^6,45^ Likewise, ketamine's effect has been identified in patients who are currently taking other antidepressant medications, as well as those who are drug-free or have completed a washout period.^6,41,46,49^

### Bipolar disorder

The majority of research on ketamine treatment for bipolar disorder has involved adults identified as resistant to prior treatment, and primarily tested ketamine as an adjunct to other treatments such as mood stabilisers or ECT.^15,56^ We identified 17 systematic reviews of the antidepressant effect of ketamine in the treatment of bipolar disorder, nine of which included meta-analyses. One review^56^ and two meta-analyses^57,58^ focused exclusively on bipolar disorder, whereas the remaining 14 also reviewed unipolar depression.

Similar to unipolar depression, the reviews found a rapid and short-lived antidepressant effects of ketamine in patients with bipolar disorder.^6,8,9,14,50,56,57^ Meta-analyses identified effects with onset as early as 4 h^43^ and consistently by 24 h.^6,9,14,50,57,58^ Findings are less consistent with respect to duration, with some analyses concluding that effects are limited to 3 days^14,57^ and others reporting that effects retain superiority over placebo at 7 days.^43,50^ Meta-analyses at 14 days did not find an effect of ketamine.^14,43,57,58^

Comparisons of effect size in unipolar versus bipolar depression populations have not yielded consistent results. A meta-analysis found no difference at 4 h after administration, and two out of three meta-analyses reported no difference at 24 h after infusion; in contrast, one meta-analysis found a larger antidepressant effect for MDD at 24 h.^9,43,50^ Similarly, equivocal results are seen at 7-day follow-up: one meta-analysis identified a stronger effect in bipolar depression^43^ and one found no difference.^50^ Notably, the seven meta-analyses included in the present review rely on data extracted from two RCTs conducted by the same research group.^59,60^ In sum, extant findings do not support a consistent difference in ketamine effects on depressive episodes in MDD versus bipolar disorder.

The main concern of ketamine treatment for bipolar disorder is the risk of inducing mania. Although manic symptoms in patients with bipolar disorder have been noted to increase immediately following ketamine infusion, these symptoms tend to resolve within 1 or 2 h.^6,14,56^ In a meta-analysis, it was reported that treatment emergent mania was not observed statistically significantly more frequently in the active intervention group compared with the placebo.^58^ However, this meta-analysis is probably underpowered to detect uncommon adverse effects such as mania.^58–60^

Likewise, evidence from case studies, open-label trials and retrospective chart analyses suggest that non-intravenously (i.e. intramuscular, intranasal and oral) administered ketamine is effective and safe for adults with bipolar disorder.^3,12,15,54,56^

### Suicidal ideation

We identified six systematic reviews of the effects of ketamine on suicidal ideation,^61–66^ two of which included a meta-analysis of studies^62,66^ and one with a meta-analysis of individual participant data.^63^ The ketamine doses ranged from 0.1 to 3 mg/kg, and were most frequently administered intravenously, although oral suspension, intranasal, intramuscular and subcutaneous bolus infusions have also been used in a number of studies.^61,62,64–66^ Two reviews were limited to trials of a single dose of ketamine,^62,63^ and the other reviews included studies of multiple doses of ketamine, but did not directly compare doses.^61,64–66^ Further data comparing the safety and efficacy of single and repeated doses of ketamine for suicidal ideation is needed.

The one review that examined route of administration found no differences between a bolus dose (0.2 mg/kg) and an infusion (0.5 mg/kg).^62^ However, there is limited evidence on intramuscular, intranasal and oral formulations for use in suicidal ideation.^65^ Additionally, in one study of intranasal esketamine, no participants managed to self-administer the ten pumps to receive the full dose, because of adverse effects; suggesting that the tolerability of intranasal administration may be poor.^66,67^

All meta-analyses and systematic reviews concluded that ketamine treatment was associated with a moderate-to-large decrease in suicidal ideation (Cohen's *d* = 0.51–0.85).^61–66^ These effects appeared within the first 4 h of treatment^61,62,66^ and were maintained for an average of 3 days^61,66^ up to a week,^63,65^ although long-term effects are not reported.^65^ Among participants who showed remission of suicidal ideation within 24 h of treatment, the treatment effect persisted for up to 1 week in approximately 85% of participants. Interestingly, ketamine effects remained significant after adjusting for change in depression, suggesting that ketamine may have anti-suicidal effects independent of antidepressant effects.^63,65^ In one meta-analysis, one of the studies included data on suicide attempts up to 28 days after treatment; no participants had attempted suicide in either the ketamine or placebo group during this time period.^66^ Interestingly, in another review it was reported that ketamine led to transient improvements in suicidal ideation in individuals who had been admitted to hospital because of a suicide attempt or in those with chronic and severe suicidal ideation.^65^ Although these data were based on case reports, this high suicide risk population has been excluded from other trials.^65^

### Interactions with ECT

The combined anaesthetic and antidepressant action of ketamine make it a logical choice for use in ECT, with the hope that it might exert additive or perhaps synergistic effects.^68^ We identified 19 studies of patients with MDD, bipolar disorder and treatment-resistant depression, in which ketamine was either used as an anaesthetic during ECT or for relapse prevention following ECT treatment. All studies used intravenous administration with doses ranging from 0.5 to 2 mg/kg. Ketamine was superior in reducing depressive symptoms in eight of the 18 studies when compared with an active control such as methohexital, thiopental or propofol.^69–82^

The most consistent evidence for effect of ketamine with ECT is in the treatment of treatment-resistant depression: three double-blind RCTs, one partially randomised trial, one open-label trial and one retrospective review have reported positive findings for the impact of ketamine on the effectiveness of ECT for treatment-resistant depression, whereas three RCTs reported no differences between ketamine and placebo.^83,84^ In general, these studies reported that, relative to other anaesthetics, ketamine was associated with earlier improvements in depression, higher remission rate, better overall improvement longer seizure duration, fewer required ECT sessions and less cognitive impairment (but see also Carspecken et al^79^).^69–71,76,78,82^

In contrast to findings demonstrating the benefit of ketamine as an adjunct to ECT for treatment-resistant depression, results from MDD and bipolar disorder are less supportive. A double-blind RCT of 31 in-patients with MDD reported a more rapid antidepressant effect, lower required electrical dose and longer seizure duration with ketamine anaesthesia during ECT.^77^ In a larger RCT with 172 patients, low-dose (0.3 mg/kg) ketamine-assisted ECT was associated with higher remission rate compared with routine ECT with propofol; interestingly, intermittent ketamine administration (one in every three ECT sessions) resulted in lower rates of psychiatric complications compared with repeated ketamine administration with each ECT session.^85^

Nonetheless, results from six other studies suggest no difference between ECT with ketamine and ECT with control conditions in reducing depressive symptoms in those with MDD or bipolar disorder,^73–75,80,81,86^ although one study was reported to lack power because of small sample size.^86^ Divergent results may be a result of differences in effectiveness for adjunctive ketamine in ECT, but may also reflect differences in treatment as the optimal dosage and frequency remains undetermined. Indeed, studies that reported no benefit for ketamine generally involved lower doses of 0.3–0.5 mg/kg,^73–75^ but not always.^86^

Aside from the use of ketamine as an anaesthetic agent during ECT, one pilot study aimed to examine the effectiveness of ketamine for relapse prevention following response to ECT.^87^ However, out of 26 patients who responded to ECT treatment, only six were willing to take part, some because of a lack of interest in further treatment following successful ECT, and none of the participants completed the full 4-week treatment protocol.^87^

### Social anxiety and generalised anxiety disorder

We identified six studies of ketamine treatment for social anxiety disorder and/or generalised anxiety disorder (GAD). A retrospective review of patients receiving multiple sessions of ketamine assisted therapy (sublingual and/or intramuscular) in a private practice setting, revealed significant decrease in anxiety following treatment compared with baseline.^12^ A study of open-label ascending dose ketamine (0.25, 0.5 and 1 mg/kg intramuscular at 7-day intervals) among 12 patients with treatment refractory social anxiety disorder and/or generalised anxiety disorder reported that 1 mg/kg was associated with the greatest duration of anxiolytic effects for up to 7 days after infusion.^34^ Following the 0.5 mg/kg or 1 mg/kg doses, 10 out of 12 patients reported a 50% or more reduction in anxiety and/or fearfulness.^34^ In a double-blind study, 12 patients with treatment-resistant generalised anxiety disorder and/or social anxiety disorder received three ascending doses of ketamine (0.25, 0.5 and 1 mg/kg) and midazolam (0.01 mg/kg) at 1-week intervals.^88^ Eight out of the 12 patients experienced at least a 50% reduction in anxiety and/or fearfulness.^88^ A subsequent study from the same investigators reported dose-related decreases in scores on measures of anxiety and fearfulness.^89^ These studies^34,88,89^ established a higher dose of ketamine (1 mg/kg) as having the greatest and most durable anxiolytic effects, although one reported dose-related improvements in fearfulness, but not anxiety symptoms.^88^

Participants who responded to ketamine treatment in the previous two studies^34,89^ were enrolled in an open-label maintenance treatment study for 14 weeks (*n* = 20), where they received repeated doses of 1 mg/kg of ketamine over 12 weeks, with the dose frequency adjusted according to the duration of response to ketamine.^35^ Patients who responded to initial ketamine treatment remained in remission with maintenance treatment over 3 months; however, the recurrence of symptoms was common within 2 weeks of stopping treatment.^35^

A cross-over RCT of a single dose of ketamine (0.5 mg/kg) for social anxiety disorder reported that ketamine led to significantly greater number of responders compared with placebo, with six out of 18 participants experiencing a reduction in clinician-rated anxiety scores of at least 35%, compared with none of 17 participants who received placebo.^90^ Similarly, 16 out of 18 participants who received ketamine experienced 50% or more reduction in self-reported anxiety scores, compared with nine out of 17 in the placebo group; this group difference was statistically significant.^90^

### Obsessive–compulsive disorders

We identified two studies. First, in a cross-over RCT, patients without depression and with chronic and treatment-resistant obsessive–compulsive disorder who received a single dose of intravenous ketamine had lower obsessive–compulsive disorder scores than those receiving placebo at 4 h and at 7 days following the infusion.^91^ Half of those receiving ketamine demonstrated a 35% or more reduction in obsessive–compulsive disorder symptoms, compared with none of the placebo group. An open-label trial of a single dose of ketamine (0.5 mg/kg intravenous) in ten participants with severe treatment refractory obsessive–compulsive disorder found a significant but transient benefit in clinician-rated obsessive–compulsive disorder symptoms at 1–3 h after infusion.^92^

### Post-traumatic stress disorder

Eight publications that investigated the association between ketamine and post-traumatic stress disorder were identified. One RCT^93^ and two open-label trials expressly focused on ketamine treatment for post-traumatic stress disorder symptoms,^94,95^ whereas the other five retrospectively examined the effects of peri-traumatic anaesthetic ketamine administration during treatment for physical injury.^96–100^

The first retrospective study found that patients who were administered ketamine following trauma exposure demonstrated increased post-traumatic stress disorder symptoms at 1 year,^96^ and a subsequent study by the same group identified increased symptoms of acute stress disorder, a precursor to post-traumatic stress disorder, 3 days subsequent to emergency treatment in people given ketamine compared with opioid and other analgesics.^97^

Three studies examining post-traumatic stress disorder in USA military personnel who had been administered ketamine for service-related injuries reported conflicting results. In the first study, receiving perioperative ketamine was associated with lower post-traumatic stress disorder prevalence (27% compared with 46%) among those who had not received ketamine, despite increased burn size, longer hospital stay and increased number of operations in the ketamine group.^98^ Ketamine use during surgical procedures also had a significant but weak negative correlation with post-traumatic stress disorder.^98^ In contrast, two subsequent studies reported comparable rates of post-traumatic stress disorder and post-traumatic stress disorder symptoms among ketamine and non-ketamine groups, although one of these studies was reported to have low power (<%80).^99,100^

Three studies focused on ketamine for the treatment of post-traumatic stress disorder. An open-label trial of six infusions (0.5 mg/kg) over 12 days in 15 military veterans with chronic post-traumatic stress disorder and depression reported a significant decrease in post-traumatic stress disorder and depression symptoms 24 h after the final infusion.^94^ At 14 days post-treatment, 80% of the sample were in remission from post-traumatic stress disorder symptoms, and 40% remained in remission at the end of the 56-day follow-up period.^94^ Similarly, in another open-label study, 30 participants received six ketamine infusions beginning at 1 mg/kg; a significant reduction (44%) in post-traumatic stress disorder symptoms was observed from baseline to before the sixth infusion.^95^ A cross-over RCT of a single intravenous subanaesthetic dose of ketamine (0.5 mg/kg) among 41 patients with chronic post-traumatic stress disorder reported that post-traumatic stress disorder symptom severity was improved compared with midazolam at 24 h, but not at 7 days.^93^

### Substance use disorders

We identified 14 studies examining ketamine as a treatment for substance use disorders, including six studies focusing on alcohol use disorder,^16,17,101–104^ five on cocaine use disorder^31,105–108^ and three on opiate use disorder.^18,19,109^

#### Alcohol use disorders

One study examined ketamine in combination with aversive therapy approaches that aim to establish negative links between alcohol consumption and alcohol's detrimental effect.^16^ It was reported that 70% of participants in the ketamine group remained abstinent at 1 year compared with 24% of participants who received aversion therapy alone.^16^ A subsequent study compared in-patients who selected KAP versus conventional psychotherapy reported 12-month abstinence rates of 66% for the ketamine group compared with 24% of controls.^17^

More than 20 years later, an RCT of a single ketamine intravenous infusion (0.71 mg/kg) paired with motivational enhancement therapy for alcohol dependence reported that ketamine significantly increased abstinence rates and led to a lower likelihood of alcohol use and heavy drinking, and longer time to relapse over 21 days after infusion, compared with midazolam.^104^ Of those available at 6-month follow-up, 75% in the ketamine group reported abstinence, whereas only 27% did so in the midazolam group.^104^

Ketamine may also have a role in the detoxification stage of treatment: three retrospective cohort studies of the safety and efficacy of ketamine for the management of alcohol withdrawal syndrome concluded that ketamine was safe.^101–103^

#### Cocaine use disorders

Three studies by a single research group have investigated ketamine administration on individuals with cocaine use disorders. An experimental study in cocaine dependent volunteers reported that a single dose of ketamine (0.41 mg/kg) increased motivation to quit cocaine and reduced cue induced craving significantly more than lorazepam at 24-h follow-up.^106^ The second dose (0.71 mg/kg) further decreased cue-induced craving for an additional day after administration.^106^ At 4 weeks, all participants reported a significant reduction in amount and frequency of cocaine use.^106^ A second report on the same participants indicated that these effects were mediated by ketamine-induced mystical experiences.^105^

In a subsequent experimental controlled study with community follow-up, a single dose of ketamine infusion (0.71 mg/kg) significantly reduced choices for cocaine self-administration versus money, compared with midazolam, among 20 cocaine-dependent individuals.^107^ The reduction of cocaine choices in the ketamine group represented a 67% reduction from baseline.^107^ Furthermore, the ketamine group reported significant reductions in cocaine use during the first 3 days of follow-up compared with the midazolam group, which dissipated at 2 weeks.^107^ A subsequent report from the same sample reported that mystical, but not dissociative, acute effects mediated efficacy.^31^

A subsequent RCT from the same research group assigned 55 cocaine-dependent patients to either ketamine (0.5 mg/kg) or midazolam (0.025 mg/kg) combined with mindfulness-based relapse prevention therapy.^108^ At the end of the 14-day study period, 48% of participants in the ketamine group (*n* = 27) remained abstinent compared with 11% in the midazolam group (*n* = 28), showing a large effect of ketamine.^108^ Craving scores were also 58% lower in the ketamine group, and at the 6-month follow-up, 44% of the ketamine group reported cocaine abstinence compared with none of the midazolam group.^108^

#### Opioid use disorders

Two RCTs from the one research group examined the use of ketamine-assisted psychotherapy for heroin use disorders.^18,19^ A trial that compared a higher dose of ketamine (2 mg/kg) to a lower dose (0.2 mg/kg) among 70 detoxified heroin-dependent individuals reported reduced craving in both groups, with a larger effect from the higher dose.^18^ Craving stayed reduced in the high-dose group at 24 months, but did not last beyond 1 month in the low-dose group. The rate of abstinence was also higher in the high-dose group at both intervals.^18^ A subsequent longitudinal study that compared single versus three sessions of KAP among 53 heroin-dependent patients reported that participants who received three sessions demonstrated a significantly higher abstinence rate of 50% compared with 22% for those who received a single session.^19^

Ketamine may also assist in withdrawal from opiates. The lone RCT in this area reported that ketamine infusion (0.5 mg/kg) was associated with less additional medication required to manage acute opiate withdrawal, but was unrelated to treatment outcome at 4-month follow-up.^109^

### Eating disorders

One open-label trial of ketamine infusion (20 mg/h for 10 h) among in-patients with eating disorder diagnoses reported that nine out of 15 patients showed a marked and sustained return to normal eating behaviour and acceptance of normal weight.^110^ Variability in follow-up intervals and a somewhat idiosyncratic dosing regimen complicate inferences regarding the duration and generalisability of effects, although these results suggest that further investigation of ketamine therapy for eating disorders is needed.

### Adverse effects and risks

Two studies explicitly reported no adverse effects^16,17^ and 19 did not formally report presence or absence of adverse effects.^48,51,53,55,61–66,76,84,88,95–100^ The remaining studies all reported adverse effects, with the most common being mild and transient increases in blood pressure that returned to baseline levels within 30–120 min.^40,72,83,87,102,106^ Tachycardia and bradycardia were also reported, particularly at higher doses of ketamine,^6,86,89,90^ and more severe cardiac effects. including intermittent atrial fibrillation and single salve of ventricular extrasystoles. Were observed in two patients with pre-existing conditions who made a full recovery.^70^

Most studies also reported transient and dose-dependent dissociative and psychotomimetic effects, including unusual thought content, visual hallucinations and conceptual disorganisation that peaked during and immediately following ketamine infusion and rapidly resolved within up to 2 h after infusion.^9,14,34,40,50,52,54,58,60,72,85,87,89,90^ Across the whole review, in two separate studies, one out of 41 and 31 patients, respectively, discontinued study participation because of dissociative or hallucinatory effects.^71,93^

Dysphoria and treatment-emergent suicidal ideation were reported in one study.^92^ Additionally, there were some reports of transient mania and hypomania that resolved by 80 min following infusion^14^ among patients with bipolar disorder who underwent ketamine infusion with^74^ or without ECT,^14,58^ although treatment-emergent mania was not observed more frequently in the ketamine group compared with the placebo.^58^ Transient increases in anxiety during ketamine infusions are also frequently reported, with symptoms typically declining within 80–120 min,^3,14,90^ although the review also included two cases of anxiety persisting up to 1–2 days after infusion.^92^

Non-dissociative effects associated with ketamine administration included mild sedation, agitation, nausea and vomiting, headaches, dizziness, blurred vision, dry or numb mouth, delirium, irritability, sensory changes, urination problems, vertigo and drowsiness; these were overwhelmingly reported to dissipate within 1–2 h of ketamine infusion.^3,6,12,14,15,35,40,41,43,47,52,58,69–72,78,82,83,85,87,89–91,93,101,103,104,108,110^

We found no reports of ketamine use/misuse following treatment with ketamine, nor is there evidence of transition from medical to non-medical ketamine use.^12,54^

### Risk of bias

The results of the risk-of-bias analysis is reported in Supplementary Appendix 1 for randomised studies, Supplementary Appendix 2 for non-randomised studies and Supplementary Appendix 3 for systematic reviews/meta-analyses. Most randomised trials were judged to be at either high risk of bias or raise some concerns in at least one domain. Most common areas of concern were selection of the reported results, randomisation and allocation concealment, deviations from intended interventions, missing data and measurement of outcome. Most non-randomised studies were also judged to be at serious risk of bias. All non-randomised studies suffered from risk of bias because of a lack of control and measurement for baseline confounding, and all except one was judged to be at serious risk of bias because of the measurement of outcome. Other areas of concern included retrospective classification of outcomes, selection bias, deviations from intended interventions and missing outcome data. According to the AMSTAR Checklist, the majority of the systematic reviews were of critically low quality. Frequent critical issues were lack of reference to a registered review protocol, and lack of risk-of-bias analysis and consideration of risk of bias in the interpretation of findings.

## Discussion

**Fig. 2:**
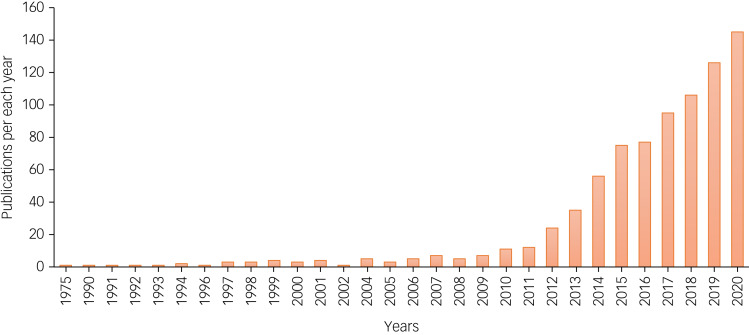
Number of publications with search terms ketamine and mental health in PubMed per year, from 1975 till December 2020.

Interest in ketamine therapies for mental health concerns expanded dramatically between 2010 and 2020 (Fig. 2). Recent FDA and European Medicines Agency approvals for esketamine in treatment-resistant depression and the broader re-emergence of psychedelic medicines prognosticate accelerating use and demand for ketamine in mental health treatment over the coming decade. The current review focused on the areas we feel are most central to clinical practice: affective disorders, suicidal ideation, anxiety disorders, post-traumatic stress disorder, obsessive–compulsive disorder, substance use disorders and eating disorders.

Affective disorders and suicidal ideation are the category of psychopathology with the most robust evidence regarding ketamine therapies, based on multiple systematic reviews and meta-analyses. Although there was some evidence to support effectiveness of ketamine administration for other indications (post-traumatic stress disorder, obsessive–compulsive disorder, anxiety and substance use disorders), the evidence base comprised a small number of mostly non-randomised trials with often short follow-up periods, therefore requiring corroboration and extension. The challenges to interpretation presented by this paucity of evidence are exacerbated by cross-study variations in dose, modes of administration and adjunctive therapies, the combination of which complicates the determination of what works for whom. Research to optimise adjunctive psychotherapy and the broader ‘set and setting’ of ketamine experiences are also required to estimate the parameters of ketamine effects in the context of psychological health. Nonetheless, extant evidence provides signals that permit some provisional observations.

Across all studies where treatment effects have been consistently observed, there is a need to profile treatment responders, along with identifying dose and route of administration and other factors that may prolong the effectiveness of ketamine therapy. Additionally, further research with comparisons to active placebos is required to evaluate comparative efficacy. Attempts to calculate the comparative efficacy of different administration routes are encumbered by the heterogeneity across studies, particularly with regards to frequency of administration: studies examining intranasal ketamine involve repeated doses, whereas most studies on intravenous ketamine consist of a single infusion.^55^ Research that explores means of extending effect duration is also required to identify best practices for ketamine in the context of psychiatric disorders. In addition to specifying optimal duration and frequency of dose, determining the extent to which clinical preparation, integration and guidance during the acute experience extends the duration and power of therapeutic effects is a research priority, as is the further elucidation of the importance of subjective effects such as mystical experiences.

From a more practical standpoint, evaluating modes of administration such as intramuscular and intranasal, which are less invasive and demanding of clinical resources, may be important for reducing barriers to access. The burden of affective disorders is borne disproportionately by those at lower levels of socioeconomic status,^111^ and as such, research should also focus on means of ensuring accessibility and equity in the delivery of ketamine therapies, including testing racemic ketamine against enantiomers. Finally, extant research does not speak conclusively to the safety of repeated administration over time, and as such, longitudinal research on safety is essential for informed estimation of risks and benefits of ketamine relative to other therapeutic options.

The combined anaesthetic and antidepressant effects of ketamine make it an appealing adjunct during ECT for depression, and our review suggests that the addition of ketamine to ECT can lead to longer seizure duration, fewer required treatment sessions, accelerate symptom reduction and time to remission. The therapeutic effects of ketamine with ECT have been most comprehensively demonstrated for treatment-resistant depression,^70,71,82^ with less robust adjunctive effects in MDD and bipolar disorder, highlighting the need for further research to clarify when and for whom ketamine might accentuate the benefits of ECT.

Given that ketamine may also be used recreationally, it is notable that even in addiction treatment, no studies in our review report a transition to illicit use engendered by introduction to ketamine in a therapeutic context. Future research should continue to monitor this potential adverse effect of ketamine therapy in the context of substance use disorders. However, given the negative consequences of substance use disorders, particularly the grave harm of overdose associated with opioid misuse, the relatively modest risks of ketamine misuse should not present a barrier to treatment for opioid use disorders or for psychiatric comorbidities that may accompany opioid misuse. Conversely, patients who use ketamine outside of medical supervision should be queried to determine the extent to which this use might reflect therapeutic motives as a drug substitute or to address negative affect.

Several areas warrant further research. In particular, the importance of subjective experience and adjunctive psychotherapy in relation to the effects of ketamine are key areas where the extant literature is limited by the lack of more rigorous randomised control designs, which have been effective in bolstering the evidence for other forms of psychedelic-assisted psychotherapy such as MDMA-assisted psychotherapy^112^ and psilocybin-assisted psychotherapy.^113^ Further research should also attempt to elucidate the mechanisms by which ketamine effects such a broad range of mental health conditions. The apparent diversity of ketamine therapeutic action has led to speculation regarding potential cross-cutting mechanisms, including the proposal that ketamine may affect the higher-order personality trait of neuroticism,^114^ enhance mindfulness and provide therapeutic experiences of self-acceptance and absence of negative emotions,^12^ but these interesting proposals have not been subject to rigorous tests.

There is also surprising paucity of research on ketamine treatment for personality disorders and eating disorders. We identified no reports on personality disorders and one small open-label trial for eating disorders. In light of recent interest in the use of psychedelic-assisted psychotherapy for eating disorders and the limited treatment options for eating disorders, further examination of the therapeutic potential of ketamine for eating disorders is warranted.^115–118^ With regard to personality disorders, the high levels suicidality associated with borderline personality makes research on ketamine for suicidality also a priority among this population. Moreover, in light of the positive effects of ketamine for depressive disorders, the lack of research on the use of ketamine in the treatment of postpartum depression is a notable gap that demands consideration.

The most common adverse effects associated with ketamine administration were increases in systolic and diastolic blood pressure, dissociation and psychotomimetic symptoms, all of which were short lived. However, given the relatively nascent state of research and practice in the area of ketamine for mental health, further research is required to definitively determine the safety and tolerability of ketamine in psychiatric populations, particularly over the longer term. Nonetheless, extant evidence on the risks of ketamine suggest a profile that is comparable to other widely used psychiatric medications.

The current review provides a comprehensive overview of the evidence base for ketamine treatment across psychiatric disorders, including depression, bipolar disorder, anxiety disorders, post-traumatic stress disorders, obsessive–compulsive disorders, substance use disorders and eating disorders. We used an extensive search strategy covering a number of databases and trial registries to identify all relevant literature. Nonetheless, there are a number of limitations to consider. First, because of the high number of studies included in the review, single review authors undertook data extraction and rated risk of bias; however, all reviewers were trained in using the forms, and a selection of data extraction and risk of bias forms were piloted for accuracy and completeness. It is also worth noting that majority of the studies included in the review were rated as at high risk of bias because of a number of methodological limitations outlined in the risk of bias results, so the results should be interpreted with caution. Moreover, although evidence from MDD suggests that ketamine effects and risks are consistent across use of concomitant medications, further research is required to assess the extent to which these apparently robust effects generalise to other conditions and across diverse classes of medications. In particular, it may be interesting to examine the extent to which ketamine effects interact with those of other interventions emerging under the banner of psychedelic-assisted psychotherapy. Additionally, included studies were heterogenous in terms of ketamine administration (dose, number, route of delivery), measurement of outcomes, length of follow-up, study design and setting, which reduces comparability.

There are a number of methodological differences between ketamine studies that would benefit from standardising to aid interpretation of findings as more psychiatric indications are researched as targets for ketamine treatment. One area of discrepancy has been the use of active (typically midazolam) versus inactive placebos (typically saline). Although earlier studies in depression used inactive placebos, active placebos have begun to be adopted. Benefits of active placebos are in providing more confidence in blinding to treatment allocation, where the subjective effects of the drug make blinding difficult, as with ketamine. Concerns about active placebos are that they may have unintended treatment consequences; for example, in studies for anxiety, where benzodiazepines are used as a treatment. However, in the studies reviewed here, this is not consistently observed; for instance, in the study by Dakwar et al,^104^ more individuals dropped out from the midazolam group and returned to heavy drinking compared with the ketamine group. Among those with anxiety disorders, midazolam had small transient effects on anxiety symptoms, equivalent to 0.25 mg/kg ketamine,^89^ suggesting that using midazolam as active control at higher doses of ketamine (e.g. 0.5 mg/kg) should not obscure the active treatment effect. In addition to using active placebos, some researchers have attempted to minimise expectancy effects by informing the participants that they may receive any one of a number of psychoactive drugs, including ketamine.^104,105^ Future trials of ketamine or other drugs with identifiable psychoactive effects can also minimise bias by having independent outcome assessors who are not present during drug administration.

Similar differences have been in the use of cross-over studies (within participants) or between-participant designs. The merit of the former is the ability to observe effects in the same participants, thus negating the issue of pre-existing group difference, but it can be confounded by persisting treatment and order effects. Similarly, with studies where participants all take part in both arms, this can compound blinding issues even where participants are given active placebos. Upon reviewing the literature, we would recommend the use of active placebos where possible, in between-participant designs.

Numerous avenues are ripe for exploration in future treatment approaches with ketamine. One area of interest is in creating trials to tackle the problem of comorbidities and multi-morbidities, which are often more representative of the psychiatric population at large, where ‘pure’ diagnoses are rare. Ketamine seems a good solution to the problem of multi-morbidities as it is emerging as an effective treatment for a number of conditions, and this raises the issue of whether strict diagnostic criteria that have been used in the past should be considered for entry into clinical trials, or whether expanding to treat presenting symptoms with a formulation-based approach may be more appropriate.

Trials have also only begun to investigate the synergies between ketamine and psychological therapy, and future research should design full factorial approaches to assess both the additive and interactive effects of combining such interventions. Based on the extant literature, it is difficult to prescribe even a minimum adequate behavioural framework for giving ketamine clinically, but research from clinical use of KAP^12^ and qualitative studies in the field^119^ suggest that sufficient preparation before the experience, a clinical and professional setting and trusting and supportive relationships with staff is crucial, as patients are very sensitive to minor disruptions in the setting. Given findings that ketamine's therapeutic benefits can be extended with psychological therapy,^29,120^ it is advisable to provide ketamine treatment alongside a psychological therapy. Certain psychological therapies may be particularly compatible with ketamine treatment. For instance, combining ketamine with motivational enhancement therapy may further increase motivation to remain abstinent in alcohol and substance use disorders, because of ketamine's effect on increasing motivation to quit drug use.^106^ Alternatively, because of ketamine's mystical and spiritual effects, it may serve to deepen the mindfulness practice cultivated by mindfulness-based therapies. However, choice of adjunctive therapy should also be informed by the symptoms of the patient population.

Another important consideration in trial design is representativeness of the trial population and access to these trials, and later therapies by individuals from disadvantaged groups. Inequalities in access to healthcare are replicated in access to clinical trials, with some exceptions.^106–108^ Trials fail to consistently report on ethnicity and socioeconomic status, which are important to gauge the generalisability of the findings to the population at large, and should be considered a minimum standard of clinical trial reporting. Recent efforts in this research space to create culturally informed research designs (e.g. Williams et al^121^) should be translated to ketamine research and are important examples of ways to diversify research in this field, an area that has been hitherto neglected and is urgently needed.^122^

In conclusion, the plethora of evidence from the systematic reviews and meta-analyses reviewed here supports a robust, rapid and transient antidepressant effect of ketamine in unipolar and bipolar depression, as well as treatment-resistant depression, with repeated dosing increasing the duration of effectiveness. In numerous studies, ketamine was also demonstrated to have short-lived anti-suicidal properties, independently of improvements in depressive symptoms. A small number of trials provide some evidence to support the beneficial effects of ketamine for post-traumatic stress disorder and obsessive–compulsive disorder. Ketamine's anxiolytic effects for social anxiety disorder and generalised anxiety disorder have also been reported, nonetheless symptom recurrence following treatment was common. There is also evidence that ketamine results in short-term increases in abstinence, reductions in use, cravings and symptoms of withdrawal related to problematic substance use. In light of the substantial mortality associated with opioid overdose, the potential of ketamine to address opioid use disorders is particularly encouraging and worthy of further investigation.

Notably, the majority of the research we reviewed was classified as being at high risk of bias, as most studies are small and have other methodological limitations. There is also much still to determine regarding synergistic effects with psychological therapy and to better specify therapeutic modalities. Research on optimal dose, route and frequency of administration that foregrounds accessibility and equity will also be paramount to reduce barriers to access among the lower income and marginalised communities that are disproportionately affected by the conditions for which this promising treatment may be effective. In sum, despite important unknowns regarding means of prolonging effects and risk over time associated with long-term repeated use, interest in the psychiatric applications of ketamine has accelerated dramatically over the past decade. This interested is warranted given ketamine's broad spectrum of potential applications in psychiatric treatment, with limited adverse effects. In light of recent progress in the development and application of ketamine-based psychiatric medicines, as well as broader developments in psychedelic-assisted psychotherapy, it seems almost certain that interest and uptake of psychiatric use of ketamine will expand even further in the decades to come.

## Data Availability

This study did not generate any new data. The template study eligibility forms and data extraction forms are available from the University of Exeter Open Research Exeter repository at http://hdl.handle.net/10871/127824.

## Supplementary material

For supplementary material accompanying this paper visit http://dx.doi.org/10.1192/bjo.2021.1061. click here to view supplementary material
