# Ketamine for the treatment of mental health and substance use disorders: comprehensive systematic review – CORRIGENDUM

**DOI:** 10.1192/bjo.2022.5

**Published:** 2022-01-18

**Authors:** Zach Walsh, Ozden Merve Mollaahmetoglu, Joseph Rootman, Shannon Golsof, Johanna Keeler, Beth Marsh, David J. Nutt, Celia J. A. Morgan

**Keywords:** Alcohol disorders, anxiety disorders, depressive disorders, mental health disorders, ketamine

In the original publication,^1^ an error was made in the introduction. In the first sentence of the Background section, ‘agonist’ should have been ‘antagonist’. This has now been corrected in both the online PDF and HTML versions of this article. The authors apologise for this error.

## References

[1] Walsh Z, Mollaahmetoglu OM, Rootman J, Golsof S, Keeler J, Marsh B, Ketamine for the treatment of mental health and substance use disorders: comprehensive systematic review. BJPsych Open, 8(1), e19. 10.1192/bjo.2021.1061. PMC8715255 35048815

