# Supplementary material for: Ketamine for the treatment of mental health and substance use disorders: comprehensive systematic review
Source: BJPsych Open. 2021 Dec 23;8(1):e19. doi: 10.1192/bjo.2021.1061 (PMC8715255; doi:10.1192/bjo.2021.1061)
Supplement: Supplementary file 1 [file S2056472421010619sup001.zip › S2056472421010619sup001.docx]

| Appendix 3 – Risk of Bias in Systematic Reviews and Meta-analyses | | | | | |  |
| --- | --- | --- | --- | --- | --- | --- |
| *Depression & Bipolar Disorder* | *Overall Quality Judgment* | *Depression & Bipolar Disorder* | *Overall Quality Judgment* | *Suicidal Ideation* | *Overall Quality Judgment* |  |
| Aan Het Rot et al., (2012) | Critically low | Alberich et al., 2017^a^ | Critically low | **Reinstatler et al., 2015** | Low |  |
| Convey et al., 2012 | Critically low | Kishimoto et al., 2016 | Critically low | **Bartoli et al., 2017** | Critically low |  |
| Katalinic et al., 2013 | Critically low | Garay et al., 2017 | Critically low | **Wilkinson et al., 2018** | Critically low |  |
| Caddy et al., 2014 | Critically low | Kraus et al., 2017 | Critically low | **D’anci et al., 2019** | Critically low |  |
| Mcgirr et al.,2014 | Critically low | Kleeblatt et al., 2017 | Critically low | **Dadiomov et al., 2019** | Critically low |  |
| Hasselmann et al., 2014 | Critically low | Papadimitropoulou et al., 2017 | Critically low | **Witt et al., 2020** | Low |  |
| Fond et al., 2014 | Critically low | Jankauskas et al., 2018 | Critically low |  |  |  |
| Serafini et al., 2014 | Critically low | Rosenblat et al., 2019 | Critically low |  |  |  |
| Lee et al., 2015 | Critically low | Zheng et al., 2020 | Critically low |  |  |  |
| Caddy et al., 2015 | High | Papakostas et al., 2020 | Critically low |  |  |  |
| McCloud et al., 2015^a^ | High | McIntrye et al., 2020 | Critically low |  |  |  |
| Romeo et al., 2015 | Critically low | Fornaro et al., 2020 | Critically low |  |  |  |
| Han et al., 2016 | Critically low |  |  |  |  |  |

Note: The AMSTAR Checklist calculates the quality of systematic/reviews on the basis of answers to 16 questions on critical and non-critical domains. The overall confidence ratings include high quality (no or one non-critical weakness), moderate quality (more than one non-critical weakness), low quality (one critical flaw with/without non-critical weakness), and critically low quality (more than one critical flaw with/without non-critical weakness). Multiple non-critical weaknesses may diminish confidence in the review. Systematic reviews solely focusing on bipolar disorder are denoted by “a”.
