# Supplementary material for: Ketamine for the treatment of mental health and substance use disorders: comprehensive systematic review
Source: BJPsych Open. 2021 Dec 23;8(1):e19. doi: 10.1192/bjo.2021.1061 (PMC8715255; doi:10.1192/bjo.2021.1061)
Supplement: Supplementary file 1 [file S2056472421010619sup001.zip › S2056472421010619sup002.docx]

| Appendix 2: Risk of Bias in Non-randomised Studies of Interventions (ROBINS-I) | | | | | | | |  |  |
| --- | --- | --- | --- | --- | --- | --- | --- | --- | --- |
|  | Bias due to confounding | Bias in the selection of participants into the study | Bias in classification of interventions | Bias due to deviations from intended interventions | Bias due to missing data | Bias in measurement of outcomes | | Bias in selection of the reported result | Overall RoB judgment |
| *OCD* |  |  |  |  |  | |  |  |  |
| Bloch et al., 2012 | Serious | Low | Low | NI | Low | | Serious | Serious | Serious |
| *GAD/SAD* |  |  |  |  |  | |  |  |  |
| Dore et al., 2019 | Serious | Moderate | Moderate | Moderate | NI | | Serious | NI | Serious |
| Glue et al., 2017 | Serious | Low | Low | NI | NI | | Serious | Serious | Serious |
| Glue et al., 2018 | Serious | Low | Low | Low | Low | | Serious | Serious | Serious |
| *PTSD* |  |  |  |  |  | |  |  |  |
| Albott et al., 2018 | Serious | Low | Low | NI | Low | | Serious | NI | Serious |
| Highland et al., 2020 | Low | Low | Serious | Low | Low | | Serious | NI | Serious |
| McGhee et al., 2008 | Serious | NI | Serious | Moderate | Low | | Serious | NI | Serious |
| McGhee et al., 2014 | Serious | Serious | Serious | Serious | Low | | Serious | NI | Serious |
| Schonenberg et al., 2005 | Serious | Low | Serious | Low | Low | | Serious | NI | Serious |
| Schonenberg et al. 2008 | Serious | Serious | Serious | Low | Low | | Low | NI | Serious |
| Ross et al., 2019 | Serious | Low | Low | Low | Low | | Serious | NI | Serious |
| *Alcohol use disorders* |  |  |  |  |  | |  |  |  |
| Wong et al., 2015 | Serious | Low | Moderate | Low | Low | | Serious | NI | Serious |
| Krupitsky et al., 1997 | Serious | NI | Low | NI | Serious | | Serious | NI | Serious |
| Shah et al., 2018 | Serious | Low | Moderate | Low | Low | | Moderate | NI | Serious |
| Pizon et al., 2018 | Serious | Low | Low | Low | Low | | Low | NI | Serious |
| *Electroconvulsive Therapy* |  |  |  |  |  | |  |  |  |
| Kranaster et al., 2011 | Serious | Moderate | Serious | Low | Serious | | Serious | Serious | Serious |
| Okamoto et al., 2010 | Serious | Low | Low | Low | Serious | | NI | NI | Serious |
| Rybakowski et al., 2016 | Serious | NI | Serious | Low | NI | | Low | Serious | Serious |
| *Eating Disorders* |  |  |  |  |  | |  |  |  |
| Mills et al., 1998: | Serious | Low | Low | NI | Low | | Serious | NI | Serious |

Note: The domain risk of bias judgments include low risk of bias, moderate risk of bias, serious risk of bias, critical risk of bias and no information on which to base a judgment about risk of bias for this domain. The overall risk of bias options include low risk of bias (the study is comparable to a well-performed RCT), moderate risk of bias (the study provides sound evidence for a non-randomised study but not comparable to a well-performed RCT), serious risk of bias (the study has some important problems), critical risk of bias (the study is too problematic to provide any useful evidence) and no information on which to base a judgment about risk.
