# Supplementary material for: Ketamine for the treatment of mental health and substance use disorders: comprehensive systematic review
Source: BJPsych Open. 2021 Dec 23;8(1):e19. doi: 10.1192/bjo.2021.1061 (PMC8715255; doi:10.1192/bjo.2021.1061)
Supplement: Supplementary file 1 [file S2056472421010619sup001.zip › S2056472421010619sup003.docx]

| Appendix 1: Revised Cochrane risk-of-bias tool for randomized trials (RoB 2) | | | | | | |
| --- | --- | --- | --- | --- | --- | --- |
|  | Bias arising from the randomisation process | Bias due to deviations from the intended interventions * | Bias due to missing outcome data | Bias in measurement of the outcome | Bias in selection of the reported result | Overall RoB Judgment |
| *OCD* |  |  |  |  |  |  |
| Rodriguez et al., 2013 | Some concerns | Low | Low | High | High | High |
| *GAD/SAD* |  |  |  |  |  |  |
| Taylor et al., 2018 | Low | Low | Low | High | High | High |
| Shadli et al., 2018 | Low | Low | Low | Low | High | High |
| Glue et al., 2019 | Low | Low | Low | Low | High | High |
| *PTSD* |  |  |  |  |  |  |
| Feder et al., 2014 | Low | Low | Low | Low | High | High |
| *Alcohol use disorders* |  |  |  |  |  |  |
| Dakwar et al., 2020 | Low | Low | Low | Low | High | High |
| Krupitsky et al., 1992 | Some concerns | High | Low | Some concerns | Some concerns | High |
| *Cocaine use disorders* |  |  |  |  |  |  |
| Dakwar et al., 2014^a^ | Low | Low | Low | Low | Some concerns | Some concerns |
| Dakwar et al., 2017^a^ | Low | Low | Low | Low | Low | Low |
| Dakwar et al., 2019 | Low | Low | High | Low | High | High |
| *Opiate use disorders* |  |  |  |  |  |  |
| Krupitsky et al., 2002 | Some concerns | High | High | Low | High | High |
| Jovisia et al., 2006 | Some concerns | High | Low | High | Some concerns | High |
| Krupitsky et al., 2007 | Some concerns | Low | Some concerns | Low | High | High |
| *Electroconvulsive Therapy* |  |  |  |  |  |  |
| Altinay et al., 2019 | Low | Low | Low | Low | Some concerns | Some concerns |
| Anderson et al., 2017 | Low | Low | Low | Low | Low | Low |
| Carspecken et al., 2018 | Low | Low | High | Low | High | High |
| Dong et al., 2020 | Some concerns | High | Some concerns | Some concerns | Some concerns | High |
| Fernie et al., 2017 | Low | Some concerns | High | Low | Some concerns | High |
| Finnegan et al., 2019 | Low | Low | Low | Some concerns | Low | Some concerns |
| Gamble et al., 2018 | Low | High | High | Low | Low | High |
| Loo et al., 2012 | Low | Low | High | Low | Some concerns | High |
| Rasmussen et al., 2014 | Some concerns | High | High | Low | High | High |
| Ray-Griffith et al., 2018 | High | Some concerns | Low | Low | High | High |
| Salehi et al., 2015 | Low | High | High | Low | High | High |
| Shams-Alizadeh et al., 2015 | Low | High | Low | Low | Some concerns | High |
| Yoosefi et al., 2014 | Low | High | Low | Low | Some concerns | High |
| Wang et al., 2012 | Some concerns | High | High | Low | Some concerns | High |
| Zhang et al., 2018 | Some concerns | Low | Low | Low | Some concerns | High |
| Zhong et al., 2016 | Low | Some concerns | High | Low | Some concerns | High |

Note: * Effect of assignment to intervention. RoB: Risk of Bias. The possible risk-of-bias judgements for each domain are a) low risk of bias, b) some concerns and c) high risk of bias. Overall low risk of bias indicates that the study is judged to be at low risk of bias for all domains, some concerns indicate that the study is judged to raise some concerns in at least one domain but is not at high risk of bias for any domain. For an overall high risk of bias, the study is judged to be at high risk of bias in at least one domain or to raise some concerns for multiple domains in a way that substantially lowers confidence in the result. a: RoB for studies reporting on the same sample was only assessed and reported once (Dakwar et al., 2014a; 2014b; Dakwar et al., 2017; 2018)
