# Supplementary material for: Ketamine for the treatment of mental health and substance use disorders: comprehensive systematic review
Source: BJPsych Open. 2021 Dec 23;8(1):e19. doi: 10.1192/bjo.2021.1061 (PMC8715255; doi:10.1192/bjo.2021.1061)
Supplement: Supplementary file 1 [file S2056472421010619sup001.zip › S2056472421010619sup004.docx]

| **Table 10:** Characteristics and results of included studies on substance use disorders | | | | | | |
| --- | --- | --- | --- | --- | --- | --- |
| **Authors, date & design** | **Patient demographics** | **Ketamine treatment** | **Control** | **Adjunct treatments** | **Outcome measures** | **Results** |
| Mills et al., 1998  United Kingdom | 15 patients with chronic and treatment-resistant Anorexia or Anorexia and Bulimia Nervosa (separate or comorbid) –  N = 5 with diagnosis of concurrent compulsive eating, vomiting or thirsting. | Dosage varied on a patient-by-patient basis.  Determined dose administered at 20 mg each hour over 10hours.  Ketamine administration range: 2 – 9 infusions. | No active/pharmacological control  Control group comprised of Coping Questionnaire scores obtained by 42 anorexic patients, for comparison. | Patients received 20 mg of nalmefene twice daily.  All patients also prescribed amitriptyline | The Coping Questionnaire (inc. Compulsive sub-scales) | 60% of patients demonstrated durable clinical remission, extending well beyond hospital discharge.  Significantly reduced compulsion scores amongst ketamine responders following treatment. (Mean P = 0.0016. However, still remained significantly elevated, relative to controls. (P = <0.001)  Compulsive Eating and Starving scores significantly decreased amongst treatment responders. (P = p=0.035 and p=0.043) |
