# Supplementary material for: Ketamine for the treatment of mental health and substance use disorders: comprehensive systematic review
Source: BJPsych Open. 2021 Dec 23;8(1):e19. doi: 10.1192/bjo.2021.1061 (PMC8715255; doi:10.1192/bjo.2021.1061)
Supplement: Supplementary file 1 [file S2056472421010619sup001.zip › S2056472421010619sup005.docx]

| **Table 9:**  Characteristics and results of included studies on substance use disorders | | | | | |  |  |
| --- | --- | --- | --- | --- | --- | --- | --- |
| **Authors, date, location & design** | **Patient demographics** | **Ketamine treatment** | | **Control** | **Adjunct treatments** | **Outcome measurements** | **Results** |
| ***Alcohol use disorders*** | | | | | | | |
| Wong et al., 2015, USA  Retrospective cohort review | 23 participants, (14 females) alcohol withdrawal syndrome | | Initial infusion dose mean: 0.21 mg/kg per hour. | N/A |  | Decrease in benzodiazepine requirements  Alcohol withdrawal symptoms | Ketamine resulted in a non-significant decrease in benzodiazepine requirements. Alcohol withdrawal symptoms resolved within a mean of 5.6 days after ketamine administration. |
| Shah et al., 2018,  Retrospective cohort review | 30 patients, severe alcohol withdrawal, | | Initial median dose of 0.75 mg/kg/h and average maximum daily infusion dose of 1.6mg/kg/h | N/A | Lorazepam (average infusion rate of 14mg/h)  Phenobarbital  Diazepam | Alcohol withdrawal symptoms, reduction in lorazepam infusion rates | Initial control of alcohol withdrawal symptoms was reached within 1 hour of ketamine administration for all patients. Within 48 hours, 43% stopped lorazepam infusions completely. |
| Pizon et al., 2018  Retrospective cohort review | 63 patients (12 females), ethanol withdrawal | | Initial mean infusion dose 0.24 mg/kg/hr  Total median ketamine dose 825 mg | N/A | Diazepam, Lorazepam, Midazolam, Benzodiazepines, Propofol, and/or Dexmedetomidine | Decrease in benzodiazepines requirements, alcohol withdrawal symptoms. | Ketamine infusion was associated with significantly reduced benzodiazepine requirements. |
| Krupitsky et al., 1997 Russia  Non-randomised study | 211 males, alcoholism with alcohol withdrawal syndrome | | Intramuscular ketamine (2.5mg/kg) | Treatment as usual with Conventional psychotherapy | Ketamine psychotherapy (KTP) | Abstinence rates at 1-year, 2- and 3-years follow-up. | 65.8% of ketamine group were abstinent at one-year vs 24% of control group. Among the ketamine group, abstinence of two years was observed in 41% of 81 patients, and abstinence of 3 years was observed for 33% of 42 patients who were available to follow-up. The control group was not followed beyond one year. |
| Krupitsky et al., 1992, Russia  Randomised controlled trial | 186 males with alcoholism | | Intramuscular ketamine (3mg/kg) | Aversive therapy | Aethimizol (1.5% 3m), Hemegride (0.5% 10 ml)  Affective Contra attribution therapy | Abstinence and relapse rates at 1 -year follow-up | At 1 year 69.8% of the ketamine group were abstinent and 27.9% relapsed. In the control group, only 24 people were abstinent (24%), the remainder were drinking alcohol. |
| Dakwar et al., 2020. USA  Randomised controlled trial | 40 participants, (21 females) with alcohol dependence | | 0.11 mg/kg over 2 min bolus followed by 0.6 mg/kg over 50 minutes. | Active control (Midazolam 0.025 mg/kg) | Motivational Enhancement Therapy | Proportion of Abstinence in control vs active group at 21 days post infusion | Over 21 days following the infusion, 47% of those in the ketamine group consumed alcohol relative to 59% in the midazolam group. 17.6% of those in the ketamine group had a heavy drinking day compared to 40.9% in the midazolam group. |
| ***Cocaine use disorders*** | | | | | | | |
| Dakwar et al.,2014a, USA  Randomised controlled cross over trial | 8 participants (1 female), dependence on crack cocaine | | 52 minutes infusions  0.41mg/kg first dose  0.71 mg/kg second dose, 48 hours between doses | Lorazepam active control | N/A | University of Rhode Island Craving Assessment, Visual analogue scale for cocaine craving, and cocaine use | The first ketamine dose compared to lorazepam significantly increased motivation for changing cocaine use (median 3.6 vs 0.15) and reduced craving (median change -126, vs 65). A subsequent ketamine dose resulted in further significant reductions in craving relative to lorazepam (median -18 vs 53), but not on motivation to change cocaine use. |
| Dakwar et al., 2017, USA  Randomised controlled cross over trial | 20 participants (9 females), cocaine dependence | | 0.11mg/kg 2 min bolus followed by 0.60 mg/kg. | 2-minute saline bolus followed by active control Midazolam (0.025 mg/kg) | N/A | Cocaine self-administration: 5 choices of 25mg free base cocaine or 11$, self-reported cocaine use | Ketamine resulted in a significant reduction of cocaine choices 28 hours after the administration compared to control (1.61 vs 4.33 choices with midazolam). This represented a 67% reduction in cocaine choices with ketamine compared to baseline. Ketamine led to reductions in cocaine use during the two weeks follow-up, however this lasted only for several days. |
| Dakwar et al.,2019, USA  Randomised controlled trial | 55 participants (14 females), cocaine dependence | | 0.5 mg/kg, slow drip 40 minutes infusion. Single dose. | Active control Midazolam (0.025 mg/kg) | Mindfulness based relapse prevention | 2 weeks end of study abstinence confirmed by urine test, relapse rates, weekly cocaine use (w2-5), weekly craving scores (w1-5) | Abstinence in ketamine group 48.2% vs to 10.7% in midazolam group at the end of treatment. In ketamine group 57.5% went on the use cocaine or drop out compared with 92.9% (26/28) in the control group. Odds of cocaine use in control group were 7.8 times the odds in the ketamine group. Craving scores were 58.1% lower in the ketamine group compared to control. |
| ***Opiate use disorders*** | | | | | | | |
| Krupitsky et al., 2002, Russia  Randomised study with no control | 70 participants, (15 females), heroin dependence | | 2.0 mg/kg ketamine infusion  Single dose. | 0.2 mg/kg ketamine infusion.  No placebo control. | KPT | Visual analogue scale of craving, abstinence rates at 2 years. | The rate of abstinence was significantly higher in the high dose group than low dose group over the 24 months follow-up (respectively over 80% vs over 50% in the first month to nearly 20% vs <5% at 24 months).  Greater reduction in craving in the high dose group was observed compared to the low dose group (mean 7.7 vs 20.2 at 1 months to mean 0.6 vs 12.5 at 18 months). |
| Krupitsky et al., 2007, Russia,  Randomised study with no control | 59 participants (10 females), heroin dependence | | Intramuscular ketamine at 2mg/kg  Single dose of ketamine. | Intramuscular ketamine at 2mg/kg  Three doses of ketamine.  No placebo control. | KPT or Addiction counselling | Relapse and abstinence rates at 12 months. | At 12 months, 50% of multiple KPT group and 22.2% of single KPT were group abstinent. |
| Jovaisa et al., 2006, Lithuania  Randomised controlled trial | 50 participants (7 females), opiate withdrawal syndrome | | 0.5 mg/kg/hr ketamine infusion.  Single dose. | Saline solution | N/A | Subjective and Objective Opiate Withdrawal Scales, clozapine and clonazepam requirements, self-reported opiate use at 4 months follow-up. | Ketamine led to less additional clozapine and clonazepam at 48 hours. At 4 months there was no significant difference in opiate use between the ketamine and control groups (respectively mean opiate free weeks 9.4 vs 8) |

Note: Dakwar et al., 2014b and 2018 were not included in the table as they report data from the same participants.
