# Supplementary material for: Ketamine for the treatment of mental health and substance use disorders: comprehensive systematic review
Source: BJPsych Open. 2021 Dec 23;8(1):e19. doi: 10.1192/bjo.2021.1061 (PMC8715255; doi:10.1192/bjo.2021.1061)
Supplement: Supplementary file 1 [file S2056472421010619sup001.zip › S2056472421010619sup006.docx]

| **Table 6:**  Characteristics and results of included studies on PTSD | | | | | |  |
| --- | --- | --- | --- | --- | --- | --- |
| **Authors, date & design** | **Patient demographics** | **Ketamine treatment** | **Control** | **Adjunct treatments** | **Outcome measures** | **Results** |
| **Schonenberg et al., 2005**  Retrospective chart review | 56 moderately injured accident victims screened for PTSD approx. 1-year post-accident. | (S)-ketamine (n=12) or racemic ketamine (n=17) during ambulance transportation immediately post-accident | Those treated with opiates only during ambulance transportation immediately post-accident (n=27) | S-ketamine and racemic ketamine paired with benzodiazepine midazolam (ataranalgesia)  Short anaesthesia for surgery or analgesics administered for severe pain during hospitalisation (typically fentanyl combined with midazolam for surgery and tramadol or piritramide against moderate to severe pain.)  3 patients in opioid condition received beta-blockers. 3 patients in the ketamine group and 8 patients in the opioid group received lormetazepam, flunitrazepam, or oxazepam once or twice against sleeplessness during hospitalisation. | Impact of Event Scale-Revised, Acute Stress Disorder Scale and Peritraumatic Dissociative Experiences Questionnaire. | Retrospective: S-ketamine group displayed significantly elevated ASDS scores (on dissociation, reexperiencing & avoidance subscales), compared to opioid group. Also, higher (though not significantly) dissociation and reexperiencing scores amongst S-ketamine relative to racemic. Racemic ketamine group displayed elevated dissociation and reexperience symptoms, relative to opioid group.  Current: S-ketamine group displayed significant heightened current PTSD symptoms (IES-R scores) compared to opioid and racemic groups. No significant differences in present symptomatology between racemic ketamine and opioid groups. |
| **Schonenberg et al., 2008**  Retrospective chart review | 50 moderately injured accident victims, screened for acute stress disorder and dissociative symptoms within 3 days of hospital admission | Those treated with a single or fractionated dose of racemic ketamine during initial emergency treatment (n=13) | Those receiving opioids (n=24) or weak grade 1-2 non-opioid analgesics (n=13) | Benzodiazepine midazolam (ataranalgesia) | Acute Stress Disorder Scale, Peritraumatic Dissociative Experiences Questionnaire and Traumatic Life Event Questionnaire. | Ketamine-exposed group displayed consistently heightened ASDS and PDEQ scores, relative to both control groups (specific effect of medication on dissociation, reexperience and hyperarousal ASDS subscales) No significant differences between control groups on any PDEQ or ASDS subscales. |
| **McGhee et al., 2008**  Retrospective chart review | 147 US military soldiers who sustained thermal injuries, had undergone at least 1 operation, and had been screened for PTSD at some point post-accident. | Those who had received intraoperative ketamine during surgery (n=119) | Those who had not received intraoperative ketamine (n=28) | IV Morphine (equivalent units calculated with standard opioid conversion calc.) | PTSD Checklist-Military | 27% prevalence of PTSD amongst participants who received ketamine, relative to 46% prevalence amongst non-ketamine-exposed group. Negative although weak relationship between PTSD and ketamine. No relationship between PTSD and morphine (equivalent units) during surgery. |
| **McGhee et al., 2014**  Retrospective chart review | 289 burned US service members who had undergone at least 1 operation, and been screened for PTSD at least 30 days post injury | Those who received intraoperative ketamine (n=189) | Those who did not receive intraoperative ketamine (n=100) | Opioids received either interoceptively or on hospital ward (converted to Morphine equivalent units mg/day) | PTSD Checklist-Military | No significant difference between prevalence of positive PTSD screening amongst ketamine-exposed group (n =28%) relative to non-exposed (n=26.15%) |
| **Highland et al., 2020**  Retrospective review | 1158 veterans with combat-related injuries. | Participants (n = 107) who received ketamine infusions upon hospitalisation following combat evacuation, ≥7 days before psychiatric assessments for PTSD. | Non-ketamine group (n = 1051) defined as those who had not received ketamine prior to hospital psychiatric assessment for PTSD. | N/A | PCL Checklist | No significant difference between probability of positive PTSD screen or symptom level between ketamine (9.35%) and non-ketamine exposed (7.48%) groups. No significant difference between PCL scores between ketamine and non-ketamine exposed groups. (M diff = 1.98) |
| **Ross et al., 2019**  Open label trial | 30 US veterans with combat-related PTSD. | X6 IV infusions, 1hour over 2-3 weeks. Initial dose 1 mg/kg body weight and adjusted over subsequent sessions achieve PTR. Final mean dosage of 164.83 mg. | N/A | N/A | PTSD Checklist | Significant reduction in PTSD symptoms (mean score reduction of 44%) |
| **Albott et al., 2018.**  Open-label trial | 15 veterans with diagnosed chronic PTSD & comorbid, moderate-to-severe, treatment-resistant depression. | 6 IV infusions, 0.5mg/kg (3x weekly across a 12-day period) | N/A | All participants continued with ongoing psychotropic medication(s) throughout study duration (eg. SNRI and ‘other antidepressants’ inc. bupropion & mirtazapine | PTSD Checklist | Ketamine treatment significantly reduced PTSD symptoms from baseline assessment, to 24hrs following final (sixth) infusion. 60% of participants remitted from PTSD following first ketamine infusion and 80% in remission following sixth infusion. Median relapse time for the 12 PTSD-remitted participants was 41 days. |
| **Feder et al., 2014**  Randomised controlled crossover trial | 41 patients with chronic PTSD | Single dose 0.5mg/kg IV, 40mins (n=22) | Single dose 0.045mg/kg midazolam (n = 19) | N/A | Impact of Event Scale-Revised for PTSD symptom severity and Montgomery-Asberg Depression Rating Scale | Significant improvement in IES-R scores within 24hours post-infusion (M diff = 12.7), relative to placebo. Greater decrease in PTSD symptoms following treatment with ketamine evident in both crossover and first-period analyses. Symptoms remained significantly reduced at 2-week follow-up in the 7 ketamine-responders, relative to 1 patient who responded to midazolam. |

| **Table 7:**  Characteristics and results of included studies on generalised and social anxiety disorders | | | | |  |  |
| --- | --- | --- | --- | --- | --- | --- |
| **Authors, date & design** | **Patient demographics** | **Ketamine treatment** | **Control** | **Adjunct treatments** | **Outcome measures** | **Results** |
| **Dore et al., 2019**  Retrospective review | 235 patients with range of DSM-V diagnoses, most common including MDD, cPTSD, ADHD, PTSD & GAD. | Either ketamine SL, ketamine IM injection or both SL & IM. 61.5% of participants administered IM ketamine at average dosage of 80-90mg. SL ketamine administered at average dosage of 200-250mg. | N/A | Ketamine Assisted Psychotherapy. Maximum of 25 in-office therapy sessions.  Participants also prescribed ongoing pharmacological treatments inc. antidepressants, mood stabilisers and antipsychotics. (mean = 2.84 medications each) | Primary measures*:* Becks Depression Inventory and Hamilton Anxiety Scale. | Clinically significant reductions in anxiety and depression scores in accordance with HAM-A and BDI scores. Intake HAM-A scores fell within ‘Moderate’ classification (M = 20.35) and reduced by 5.5 on average to the mild-anxiety category. |
| **Glue et al., 2017**  Non-randomised, open label, within-subjects ascending dose study | 12 patients with treatment refractory Generalised Anxiety Disorder and/or Social Anxiety Disorder (without co-morbid depression) | 0.25, 0.5, and 1mg/kg subcutaneous, ascending | N/A | Participants remained on prescribed medication and continued with psychotherapy where ongoing (no adjustments to dosage or therapy schedule) | Fear Questionnaire and HAM-A scale. | 83% of participants demonstrated reduction in HAM-A and/or Fear Questionnaire scores following 0.5 or 1mg/kg doses. Within 1 hour of infusion, patients reported reduced anxiety, which persisted for up to seven days. |
| **Glue et al., 2018**  Open label study | 20 patients with treatment-refractory GAD or SAD. | One or two weekly subcutaneous infusions, 1mg/kg across 3-month period. | N/A | Participants remained on prescribed medication and continued with psychotherapy where ongoing (no adjustments to dosage or therapy schedule) | Fear Questionnaire and HAM-A. | Progressive reduction in baseline Fear Questionnaire scores (asymptote reached by 7.5 weeks) At 1 h post-dosing, all ratings decreased by 50%, with no further decrease at 2 h post-dose. Baseline HAM-A reached asymptote by 3.5 weeks. At 1hr post-dosing, all ratings reduced by ~50%, with no further reduction noted 2hrs post-dose. |
| **Glue et al., 2019**  Randomised, placebo-controlled ascending dose study | 12 patients with treatment refractory Generalised Anxiety Disorder and/or Social Anxiety Disorder (without co-morbid depression) | 0.25, 0.5, and 1mg/kg subcutaneous, ascending | 0.01 mg/kg midazolam subcutaneous, randomly inserted into ascending dose sequence | Participants remained on prescribed medication and continued with psychotherapy where ongoing (no adjustments to dosage or therapy schedule) | Fear Questionnaire and HAM-A. | Dose-associated reductions in  >50% reduction in HAM-A scores and/or FQ scores observed within 67% of sample following 0.5 and/or 1 mg/kg doses. Improvement in anxiety reflected within first hour post-infusion and sustained for up to 1 week. Effect of midazolam control on FQ scores overlapped with 0.25 mg/kg and 0.5 mg/kg ketamine doses. |
| **Taylor et al., 2018**  Randomised, placebo-controlled crossover trial | 18 patients with Social Anxiety Disorder. | Single dose 0.5mg/kg IV, 40mins | Single dose saline | Ongoing pharmacological treatment (SSRI’s SNRI’S or Benzodiazepines) | Liebowitz Social Anxiety Scale and VAS-Anxiety. | Significantly greater decrease in LSAS score amongst ketamine group, relative to control, though no S.D on VAS. 33.33% treatment response in ketamine group within 2weeks post-infusion, compared to 0% response following placebo (in accordance with LSAS) |
| **Shadli et al., 2018**  Randomised, controlled placebo cross over trial | 12 patients with refractory GAD and/or SAD. | 0.25, 0.5, and 1 mg/kg,  ascending. | 0.01 mg/kg midazolam. | Patients remained on their current medication regimens (eg. SSRI’s, TCA’s) and continued with psychotherapy (eg. CBT)  However, no patients started new treatments and did not adjust doses/therapy frequency. | Fear Questionnaire and HAM-A. | 67% of patients reported >50% reduction in FQ and/or HAM-A scores following the 0.5- 0r 1- mg/kg ketamine doses at 2-hour post-dose assessment. Reduction in HAM-A score did not reach significance. Dose-dependent decrease (max at 0.5mg) in theta wave frequency within right F4 area, which was the only significant predictor of improved FQ score. |

| **Table 8:**  Characteristics and results of included studies on OCD | | | | | | |
| --- | --- | --- | --- | --- | --- | --- |
| **Authors, date & design** | **Patient demographics** | **Ketamine treatment** | **Control** | **Adjunct treatments** | **Outcome measures** | **Results** |
| **Bloch et al., 2012**  Open label study | 10 patients with severe treatment refractory OCD | Single dose 0.5mg/kg IV, 40mins | N/A | 7 participants prescribed SRI’s (eg. citalopram & fluvoxamine) 4 out of this 7 taking also taking antipsychotic medication and 1 participant taking n-acetylcysteine and riluzole glutamate-modulating agents | Yale-Brown Obsessive-Compulsive Scale, Hamilton Depression Rating Scale, Clinical Global Impression scale. | Statistically significant acute improvement in OCD symptoms over initial 3 days post-infusion. However, 0% response to ketamine treatment (defined as 35% improvement in Y-BOCS at any stage across initial 1-3day period post-infusion. Statistically sig. improvement in OCD symptoms 1-7 days post-infusion, although only <12% overall, relative to 60% symptom reduction within the initial 1-hour period post-infusion (suggests only transient effect) |
| **Rodriguez et al., 2013**  Randomised controlled crossover trial | 15 outpatients with a principle diagnosis of OCD | (x2) Single dose 0.5mg/kg IV, 40mins (n=8) | Single dose placebo saline (n=7) | N/A | OCD-VAS and Yale-Brown Obsessive-Compulsive Scale. | Significant improvement in OCD-VAS score amongst ketamine group during infusion, relative to controls. At one -week follow-up, 50% of participants in ketamine group reached criteria indexing ‘treatment response’ (≥35% Y-BOCS reduction), whereas 0% of controls met ‘response’ criteria. |
