# Supplementary material for: Ketamine for the treatment of mental health and substance use disorders: comprehensive systematic review
Source: BJPsych Open. 2021 Dec 23;8(1):e19. doi: 10.1192/bjo.2021.1061 (PMC8715255; doi:10.1192/bjo.2021.1061)
Supplement: Supplementary file 1 [file S2056472421010619sup001.zip › S2056472421010619sup007.docx]

| **Table 5:**  Characteristics and results of included studies on ketamine as anaesthetic for ECT | | | | | | |
| --- | --- | --- | --- | --- | --- | --- |
| **Authors, date & design** | **Patient demographics** | **Ketamine treatment** | **Control** | **Adjunct treatments** | **Outcome measures** | **Results** |
| Kranaster, Kammerer-Ciernioch, Hoyer and Satorius., 2011  Retrospective study | 42 participants experiencing treatment resistance depression, with 16 in the ketamine group and 26 in the group | Mean dose of 46.7mg (SD:12). Number of doses varied by patient depending on response to ECT, with the mean number of doses of 8.9 | Active control: thiopental 236mg. | ECT as primary adjunct. | Hamilton Depression Rating Scale | Depression scores were significantly lower after ECT treatment with ketamine.  Fewer ECT sessions were also required in the ketamine group relative to thiopental group. |
| Okomoto et al., 2010  Non-randomized trial with control | 31 patients with treatment resistant depression.  11 received ketamine (5 men and 6 women). 20 received propofol (10 men and 10 women) | 0.8 mg/kg of ketamine IV bolus. 8 infusions | 0.8 mg/kg of Propofol IV bolus. | ECT as primary adjunct.  1 mg/kg of Succinylcholine (muscle relaxant) | Hamilton Depression Rating Scale (HDRS) | Depression scores improved earlier for ketamine group, with decreases in HDRS scores significantly greater than following propofol treatment  Observed in the 2nd and 4th ECT sessions, however no significant difference in HDRS scores after 6^th^ and 8th sessions. |
| Rybakowski et al., 2016  Non-randomised trial with control | 45 patients with drug-resistant depression | 1-1.5 mg/kg of Ketamine. Number of doses ranged from 2-5. | 2-3 mg/kg of thiopental | ECT as primary adjunct.  1-1.5 mg/kg of succinylcholine (muscle relaxant) | Hamilton Rating Scale for Depression | Depression severity was significantly reduced following final session, specifically in the 2nd experimental group (5 ketamine infusions) relative to control group. |
| Loo et al., 2012  Randomized, placebo-controlled, double-blind trial | 51 participants with major depressive disorder.  Ketamine group: 11 females (50%). Placebo group: 17 females (71%). | 0.5mg/kg of ketamine administered intravenously. 1-10 doses. | 0.5 mg/kg of saline administered intravenously. | ETC as primary adjunct. | Montgomery-Asberg Depression Rating Scale (MADRS) | No significant difference found between the ketamine and control groups on depressive symptoms. |
| Anderson et al., 2017  Randomized, double blind parallel-group trial | 79 participants with unipolar or bipolar depression.  Ketamine group: 22 females (67%). Saline group: 22 females (60%). | 0.5mg/kg of ketamine intravenously. Administered twice weekly, duration of treatment course varied on individual basis. | 0.5mg/kg of saline. | ECT as primary adjunct.  Suxamethonium (muscle relaxant) | Montgomery-Asberg Depression Rating Scale  (MADRS). | There was no evidence found to suggest a benefit of ketamine as an adjunct to ECT given at 0.5mg/kg on efficacy outcomes in patients with depression. |
| Rasmussen et al., 2014  Single-blind Randomized controlled trial | 38 participants with unipolar or bipolar depression (24 females and 14 males) | 1.0mg/kg of ketamine. Dosage was modified accordingly on a case by case basis (N = 21). 6 doses. | 1.0mg/kg of Methohexital (n=17) | ECT as primary adjunct  Glycopyrrolate (anti-sialogogue) and succinylcholine (muscle relaxant)  Intra-procedural medication (eg. labetalol and esmolol) | Patient Health Questionnaire, Hospital anxiety and depression scale. | Ketamine as an anaesthetic was not associated with an increase in the antidepressant effect of ECT. Furthermore, there was no significant differences in recovery time between the two groups. |
| Salehi et al., 2015  Single-blind randomized controlled trial | 160 patients with drug-resistant major depression. | 0.8mg/kg of ketamine intravenously. 8 doses. | 1-1.5mg/kg of thiopental intravenously. | ECT as primary adjunct. | Hamilton Depression Rating Scale. | Both ketamine and thiopental resulted in reduced depressive symptoms following each ECT session across both groups. However, reductions in symptoms were significantly greater amongst ketamine group relative to thiopental. |
| Yoosefi et al., 2014  Randomized double-blind controlled trial | 31 inpatients with a diagnosis of major depressive disorder. | 1-2mg/kg of ketamine. 6 doses. | 2-3mg/kg of thiopental | ECT as primary adjunct. | Hamilton Rating Scale for Depression. | Patients experienced earlier improvements in depressive symptoms when compared with thiopental group. |
| Zhong et al., 2016  Randomised double-blind controlled study | 90 patients with major depression or bipolar disorder with current major depressive episode. | 0.8mg/kg of ketamine. 8 doses. | Control 1: 0.8mg/kg of Propofol  Control 2:  ketamine (0.5mg/kg) plus Propofol (0.5mg/kg) | ECT as primary adjunct.  All groups also administered 1mg of Atropine Sulfate | Hamilton Depression Rating Scale. | More rapid antidepressant effects and higher remission rate observed in the ketamine-alone and ketamine plus propofol groups compared to propofol control group. Significantly elevated response and remission rates amongst ketamine-alone and ketamine plus propofol groups, relative to propofol-alone. |
| Carspecken et al., 2018  Randomised controlled trial | 50 Veterans with diagnosis of major depressive episode and defined as having treatment resistant MDD. | 1-2 mg/kg of IV racemic ketamine. Administered thrice weekly for the duration of treatment.  Length of treatment determined by clinical response. | 1-2 mg/kg of IV methohexital | ECT as primary adjunct.  All patients administered 30mg of IV ketorolac prior to general anaesthesia. | Patient Health Questionnaire and Hamilton Rating Scale for Depression. | No significant differences between changes in depression scores for ketamine and control groups. |
| Ray, Griffith et al., 2017  Randomised controlled pilot study. | 16 patients with primary diagnosis of unipolar or bipolar depression. | 1 mg/kg of IV ketamine. (N =8). 1-6 doses. | IV methohexital, 1 mg/kg. (n=8) | ECT as primary adjunct. | Hamilton Depression Rating Scale and Becks Depression Inventory. | No significant difference between response to ECT in depression scale scores as a function of treatment group (50% of ketamine group vs 25% in methohexital group) |
| Shams et al., 2015  Randomised double-blind controlled trial. | 22 patients with MDD. | 0.3 mg/kg of IV ketamine. 4 doses. | 5ml of Saline and 1 mg/kg of Propofol | ECT as primary adjunct.  0.5mg of Atropine, 1 mg/kg of propofol and 0.5 mg/kg of IV succinylcholine | Hamilton Depression Rating Scale. | Reductions in depression observed across groups, however no significant difference in changes in HDRS scores, or rate and speed of recovery between ketamine and propofol group. Findings replicated at 2-week follow-up. |
| Wang et al., 2012  Randomised double-blind controlled trial. | 48 patients with HDRS score > 20 | 0.8 mg/kg of IV ketamine. (n=16). Single dose. | Control 1: Propofol, 1.5 mg/kg (n=16)  Control 2: 0.8 mg/kg of ketamine, plus 1.5 mg/kg of Propofol (n=16) | ECT as primary adjunct.  1 mg/kg of succinylcholine (muscle relaxant) | Hamilton Depression Rating Scale | Reduction in HDRS scores observed across all groups over 1-7 days post-treatment. Decrease significantly greater amongst ketamine- alone and ketamine plus propofol groups, relative to propofol-alone control. |
| Finnegan et al., 2019  Randomised controlled pilot trial. | 6 participants with unipolar depression. | 0.5 mg/kg of ketamine over 40mins. 4 doses. | 0.045 mg/kg of Midazolam in 50ml of Saline, over 40mins | ECT as primary adjunct. | Hamilton Depression Rating Scale | No patients completed 4-week trial procedure.  Protocol deemed unfeasible. |
| Altinay et al., 2019  Randomised double-blind placebo-controlled trial. | 12 patients with treatment-resistant depression (83% female) | 0.5 mg/kg of IV ketamine over 40mins. 6 doses. | 0.0045 mg/kg of Midazolam over 40mins. | ECT as primary adjunct.  Patients able to continue existing pharmacological treatment (eg. fluoxetine and amitriptyline) | Hamilton Depression Rating Scale. | No significant difference between changes in depression scores between ketamine and midazolam groups.  42% of ketamine patients displayed early response, relative to 0% of midazolam patients. This between-group difference was significant. |
| Dong et al., 2019  Randomised controlled trial. | 134 patients with unipolar MDD. | 0.3 mg/kg. of ketamine, either repeated for each of the 6-15 ECT sessions or intermittent, once a week during the course of ECT. | Routine ECT anaesthesia with propofol (1-1.5mg/kg) and suxamethonium chloride (0.8-1 mg/kg) | ECT as primary adjunct.  All groups also received propofol, 1-1.5 mg/kg and 0.8-1 mg/kg suxamethonium chloride. | Hamilton Depression Rating Scale | HDRS scores amongst both ketamine groups were lower relative to control group, with no significant difference between the two ketamine groups.  Number of ECT sessions required to achieve remission was reduced for both ketamine groups, relative to control group. (P = < 0.01). Remission rates were also elevated amongst both ketamine groups relative to controls (p<0.001)  No significant difference in clinical cure rate across 3 groups. |
| Zhang et al., 2018  Randomised double-blind controlled trial. | 77 inpatients (41 MD and 36 BP) | 0.5 mg/kg of ketamine plus 0.5 mg/kg of propofol. (n=43). 6 doses | 1 mg/kg of Propofol (n=34) | ECT as primary adjunct.  Both groups also administered 1mg of Atropine Sulfate and 1 mg/kg of Succinylcholine (muscle relaxant | Hamilton Depression Rating Scale and Montgomery-Asberg Depression Rating Scale | Improvements in depressive symptoms observed across both ketamine and control group, although no significant difference between groups in proportion meeting response nor remission criteria. |
| Gamble et al., 2018  Randomised double-blind controlled trial | 27 patients with treatment-resistant major depressive disorder | 0.75 mg/kg  of IV ketamine. (N = 14). Number of doses varied on individual patient basis depending on response to ECT | 1 mg kg  of Propofol (n = 13) | ECT as primary adjunct.  Both groups also administered 1 lg/kg of remifentanil and 0.75 mg kg of succinylcholine (muscle relaxant) | Montgomery-Asberg Depression Rating Scale. | Adjunctive ketamine resulted in more rapid reductions in depressive symptoms relative to propofol, with a 50% decrease in MADRS scores observed following a mean of 2 sessions, relative to 4, amongst the control group. (P = < 0.01)  100% of ketamine patients obtained 50% reduction in MADRS scores (primary outcome) relative to 83% in propofol group. |
| Fernie et al., 2017  Randomised, double-blind, parallel-design controlled trial. | 40 patients with major depression although no comorbid psychiatric diagnoses | IV ketamine  Maximum dosage: 2.0 mg/kg  Twice weekly ECT sessions. Number of ketamine doses varied on individual patient basis depending on response. | IV Propofol  Maximum dosage: 2.5 mg/kg | ECT as primary adjunct.  Both groups also administered 0.5 – 1.0 mg/kg of suxamethonium (muscle relaxant) following infusion. | Hamilton Rating Scale for Depression. | No significant differences in changes in depression scores or number of required ETC sessions as a function of treatment group – Although patients in the ketamine group showed a slightly elevated improvement in HRSD scores relative to propofol group |
