# Supplementary material for: Ketamine for the treatment of mental health and substance use disorders: comprehensive systematic review
Source: BJPsych Open. 2021 Dec 23;8(1):e19. doi: 10.1192/bjo.2021.1061 (PMC8715255; doi:10.1192/bjo.2021.1061)
Supplement: Supplementary file 1 [file S2056472421010619sup001.zip › S2056472421010619sup008.docx]

| **Table 4:** Characteristics and results of included studies on suicidal ideation | | | | |
| --- | --- | --- | --- | --- |
| **Authors, date, location & design** | **Eligibility criteria** | **Summary measure & Synthesis of results** | **Study characteristics** | **Results:** |
| Dadiamov et al., 2019, USA  Systematic review | Studies published in the English language, on human subjects were included. Studies that were already included in meta-analyses, studies that did not assess suicidality or self-injurious behaviour, or those that were post-hoc analyses of included studies were excluded. All study designs were included. | Relevant items of the Hamilton Depression Rating Scale, Montgomery-Asberg Depression Rating Scale, The Quick Inventory of Depressive Symptomatology Self-Report, Beck Depression Inventory, Beck Scale for Suicidal Ideation, The Intersept Scale for Suicidal Thinking, Beck Hopelessness Scale  Narrative synthesis | 24 studies that included 2 meta-analyses, 9  clinical trials, 12 case series/reports, and 1 retrospective chart review. Sample size ranged from 1-167.  Patients were individuals with treatment resistant depression, major depressive disorder, bipolar disorder with/without suicidal ideation, suicidal attempt, obsessive compulsive disorder, paediatric Bipolar Disorder, ketamine dependence  Ketamine dose ranged from 0.2 mg/kg to 3mg/kg, or total dose of ketamine up to 165mg | The studies reviewed provide evidence for the use of IV ketamine (0.5mg/kg) to treat suicidal ideation in patients with bipolar disorder or major depressive disorders. The effects are rapid, however short lived with a typical duration of one week. Long term effects are not reported in the literature.  There is less data to support other methods of administration such as intramuscular, intranasal, sublingual for use in suicidal ideation or self-injurious behaviours. |
| Bartoli et al., 2017. Italy/London  Systematic review and meta-analysis. | Included trials of adults with current suicidal ideation from any inpatient or outpatient setting, changes in suicidal ideation measured within 4 hours of treatment. Excluded studies on treatment resistant depression, bipolar depression and those measuring suicidal ideation changes later than 4 hours of ketamine administration. | Baseline and endpoint mean scores on suicidal ideation or relevant paired t-tests to estimate standardised mean differences with 95% CI.  Individual standardised mean differences (SMD) were pooled in meta-analyses using random effects model. | 5 studies included, all conducted in the USA. Sample size ranging from 10-49, total participants included in the meta-analysis is 99.  Design of included studies was not reported.  Participants were individuals with treatment resistant depression and suicidal ideation, those with suicidal ideation or recent suicidal attempt.  Ketamine dose was 0.5mg/kg in three studies and 0.2mg/kg in two studies. | There was a large and consistent effect of IV ketamine for the acute treatment of suicidal ideation at 4 hours endpoint (SMD: -0.92, 95% CI: -1.40 to -0.44; p<0.001)  There were no statistical differences (p=0.27) between the effect of ketamine bolus (SMD=-2.11; p=0.006) vs the effect of ketamine infusion (SMD: -0.86; p=0.001) |
| Reinstatler et al., 2015  Systematic review  USA | English language, suicidal ideation at baseline, publication in a peer reviewed journal, no restriction on study type | Relevant items of the Hamilton Depression Rating Scale, Montgomery-Asberg Depression Rating Scale, The Quick Inventory of Depressive Symptomatology Self-Report, Beck Depression Inventory, Beck Scale for Suicidal Ideation.  Narrative synthesis | 9 studies were included, 3 RCTs, 3 open label non-randomised trials, 3 case reports.  Sample size of included studies was not reported.  Patients had major depressive disorder, treatment resistant depression with/without previous suicide attempts, bipolar with current depressive episode and ED patients with suicidal ideation.  Ketamine doses ranged from 0.2mg/kg to 0.5mg/kg. | Among the studies included in the review, ketamine was consistently associated with a reduction in suicidal ideation observed within 40 minutes and lasting up to 5 days. Similar findings were reported in RCTs, open label studies and case studies. |
| Wilkinson et al., 2018,  Systematic review and individual participant data meta-analysis.  USA | Studies of single dose intravenous ketamine for any psychiatric disorders were included. Comparison intervention trials were also included. Studies using multiple doses of ketamine were excluded. | A general linear mixed model (GLMM) was used with suicidal ideation as the dependent variable.  Individual patient level data analysis conducted. | 10 studies were included. Individual patient level data was obtained for 298 patients of which 167 met the criteria for baseline suicidal ideation.  Participants had a diagnosis of MDD, PTSD, BD or any psychiatric disorder.  Ketamine dose not specified. | Clinician administered outcomes:  Relative to the control group, ketamine reduced suicidal ideation with moderate to large effect sizes observed within one day (Cohen’s d=0.85 95% CI: 0.53-1.17) that extended to one-week post ketamine administration (Cohen’s d=0.61, 95% ci: 0.27-0.94).  Self-reported outcomes:  Ketamine had a moderate-to-large effect size on self-reported suicidal ideation at day 1 (Cohen’s d=0.73, 95% CI:0.38-1.07) to day 7 (Cohen’s d=0.48, 95% ci 0.12-0.83). |
| D’Anci et al., 2019  Systematic review | Nonpharmacological and pharmacological interventions for adults at risk for suicide. Systematic reviews and RCTs were included. | Scale for Suicidal Ideation, relevant items of the Montgomery-Asberg Depression Rating Scale, the Hamilton Depression Rating Scale the Quick Inventory of Depressive Symptomatology Self-Report, and the Beck Depression Inventory.  Narrative synthesis | 1 systematic review and 2 RCTs investigating ketamine efficacy to reduce suicidal ideation were included. Sample size ranged from 19-167.  Ketamine dose was 0.5mg/kg  Participant diagnoses were not reported in the review. | In all three studies included that focused on ketamine, ketamine was found to reduce suicidal ideation compared to placebo or midazolam with minimal adverse effects. |
| Witt et al., 2020  Systematic review and meta-analysis | Included randomised controlled trials of ketamine or esketamine as main or adjunct treatment with either placebo or active comparison among adults with any psychiatric disorder. | Standardised mean differences were calculated for continuous outcome data. For binary outcome data, pooled odds ratio (OR) were calculated.  The DerSimonian-Laird random-effects model was used to synthesise both continuous and categorical outcomes. | 15 trials were included. Study sample sizes ranged from 15-80 with total participants from included studies adding up to 572.  Participants had a diagnosis of unipolar depression, bipolar depression, and any mood or anxiety disorder.  The ketamine dose ranged from 0.27mg/kg to 1.0mg/kg, with 0.5 mg/kg being the most common dose. In a single esketamine trial, the total dose was 84mg. | Ketamine resulted in a significant reduction in suicidal ideation as early as 4 hours (SMD = −0.51; 95% CI =−1.00 to −0.03), at 12 to 24 hours (SMD = −0.63; 95% CI =−0.99 to −0.26), and 24 to 72 hours (SMD = −0.57; 95% CI =−0.99 to −0.14). However, ketamine treatment effect from 72 hours to 2 weeks was no longer significant. |
