# Supplementary material for: Ketamine for the treatment of mental health and substance use disorders: comprehensive systematic review
Source: BJPsych Open. 2021 Dec 23;8(1):e19. doi: 10.1192/bjo.2021.1061 (PMC8715255; doi:10.1192/bjo.2021.1061)
Supplement: Supplementary file 1 [file S2056472421010619sup001.zip › S2056472421010619sup009.docx]

| **Table 3:** Characteristics and results of included studies on bipolar disorder | | | | |
| --- | --- | --- | --- | --- |
| **Authors, Date, Location & Design** | **Eligibility criteria:** | **Summary measure & Synthesis of results:** | **Study characteristics:** | **Results:** |
| Alberich et al., 2017,  Spain.  Systematic Review | Prospective works, cases or clinical trials published in English or Spanish language.  Studies undertaken on humans only and publications included patients with diagnosis of BD | Summary measures:  Hamilton Rating Scale for Depression, or Montgomery-Asberg.  Synthesis: Narrative synthesis. | 10 studies; 1 clinical trial, 5 cohort studies and 1 case report.  Ketamine dosage ranged from 0.5 mg/kg, 50-100mg, 10 mg/kg  IV infusion was the selected method in 60% of studies. 1 study incorporated 10 mg dose of sublingual. | The literature reviewed suggests ketamine to be a viable (effective and safe) treatment for bipolar depression. However, the efficacy of ketamine is suggested to be short-term only. |
| Fornaro et al., 2020  Italy, Canada, Australia, Singapore & Spain.  Systematic Review and Exploratory Meta-Analysis. | Studies of a controlled-randomised clinical trial nature, published in peer- reviewed journals.  Studies including a minimum of 75% cases of Treatment-resistant acute bipolar depression at study baseline. Studies including pharmacological and neuromodulatory interventions or combinations. | Summary measure: Efficacy of TRBD treatment and rates of response (50% decrease in symptom scores relative to baseline, as measured by standard rating scale (inc. Hamilton-Depression Rating Scale, Montgomery-Asberg, Inventory of Depressive Symptomatology or Young Mania Rating Scale)  Synthesis: Narrative synthesis and Random effects model meta-analysis. | 17 studies in narrative synthesis. (n=928) with 3 cross-over RCTs and 9 in quantitative analysis.  Participants had acute BD (n=803) or TRBD-MA (treatment resistant bipolar depression with acute mania) (n = 125)  Ketamine dosage: 0.5 mg/kg. Number of doses not specified. | Significant reduction in depressive symptoms when ketamine was used in  augmentation of lithium or valproate – though restricted to 3^rd^ day following infusion.  Significant effect of ketamine on Day 1 following infusion, with TRBD-De ketamine-exposed patients displaying higher response rate.  (OR = 10.682 (95% C.I. = 2.142-53.272; P = <.005)  Pooled SMDs after 2 weeks did not support post-acute effect of single ketamine infusion. SMD = 0.352; 95% C.I. = - 0.135-0.840; P = .156) |
| McCloud et al., 2015  UK, Israel & Italy  Meta-analysis | Double-blind or single-blind randomised controlled trials published or unpublished. Comparing ketamine and other glutamate receptor modulators with other active psychotropics or saline placebo.  Participants were patients with primary diagnosis of Bipolar Disorder and in present depressive phase. | Summary measure:  Incidence of treatment response (defined as minimum of 50% decrease in symptoms relative to baseline, as assessed by Hamilton Rating Scale, Montgomery-Asberg or any another valid depression scale.  Number of patients who remitted in accordance with HRSD and MADRS, QIDS or IDS thresholds. | 2 studies incorporating ketamine (n = 33), used as add-on drug to mood stabilisers, with cross-over design.  Ketamine dosage: 0.5 mg/kg IV infusion. 1 – 2 doses.  Participants who were unresponsive to mood stabilisers (lithium or valproate) for 4 weeks minimum.  All inpatients and medications maintained throughout trial. | Significant effect of ketamine treatment in terms of response, relative to placebo at 24 hours post-infusion/s. (OR = 11.61; 95% C.I. = 1.25-107.74; P = 0.03)  At 72 hours post-infusion/s, effect sizes still reflected superior effect of ketamine, though not significant. (OR = 8.24; 95% C.I. = 0.84-80.61; P = 0.07)  No responders/remitters observed in either study following 2weeks post-infusion/s.  No evidence of superior effect of ketamine in remission rates, relative to placebo. |
| Lee et al., 2015.  USA.  Systematic Review and Meta-Analysis | Placebo-controlled double-blind studies published in the English language. Randomised, controlled trials of ketamine on patients with MDD or BP.  Studied including exclusively adult out-patients, diagnosed with standardized scales and included those studies in which patients continued antidepressant medication. | Summary measure: Montgomery-Asberg, Hamilton-Depression Rating Scale or Becks Depression Inventory  Synthesis: Narrative synthesis and Random effects model meta-analysis | 3 studies included BPAD I or II patients (N total = 34). All randomised, crossover, double-blind, passive placebo-controlled trials.  Ketamine dosage: 0.5 mg/kg IV ketamine, over 40mins or Saline placebo. Single dose | Ketamine appears to have robust effect on depressive symptoms among BP patients.  Day 1 post-infusion Effect size of 1.29 (C.I. = 0.75-1.89; P = <0.001)  Day 7 post-infusion Effect size of 0.28 (C.I. = 0.21-0.76; P = <.26, no effect)  Anti-depressant effect maintained at 7day post-infusion. (SMD = 0.41; 95% C.I. = 0.14-0.68; P = .003) |
| Aan Het Rot et al, 2012  Netherlands & USA.  Systematic Review | Studies including patients who met clinical criteria for a mood disorder, were in receipt of ketamine to study its antidepressant effects and were assessed clinically for at least 230 min (~4 hours) post-ketamine treatment. Also reviewed recently-completed studies, those currently in recruitment stage or not yet recruiting. | Summary measure:  Becks Depression Inventory, Hamilton Rating Scale and Montgomery-Asberg  Synthesis:  Narrative synthesis | 5 studies included BD patients. 2 RCTs (N range = 31) and 3 case studies. (N total = 3)  Patients with comorbid anxiety disorder and >1 failed trial with lithium/valproate &  Ketamine dosage: IV at 0.5 mg/kg over 40mins or IM at 0.5 – 1 mg/kg.  1 – 3 doses | Antidepressant response observed across studies between 24hours and 72 hours post-infusion in as many as 79% of patients.  Antidepressant effect sustained for up to 2 weeks post-treatment.  One case study only observed response at 24hours following ketamine infusion at dosage of 1.0 mg/kg specifically. |
| Kraus et al., 2017  Austria.  Systematic Review | Limited to clinical trials on unipolar or bipolar depression, published in peer-reviewed journals, using DSM-IV or similar assessment criteria, only standardized or reliable measures used. | Summary measure:  Hamilton Rating Scale for Depression and/or Montgomery-Asberg  Synthesis:  Narrative synthesis | 7 RCT studies assessing BD.  Ketamine dosage: IV at 0.5 mg/kg over 40mins.  Number of doses: unspecified. | All 7 studies demonstrated an antidepressant effect. Response rates reported ranged from 52%-79% (observations of a >50% decrease in HAM-D scores.) |
| Hasselmann et al., 2014  The Netherlands.  Systematic review | Duration of clinical assessment required to be >30min following treatment.  Patients to have received formal diagnosis of mood disorder and studies limited to humans. | Summary measures:  Hamilton Rating Scale for Depression  Synthesis:  Narrative synthesis | 3 studies assessed BD or MD and BD. (N total = 19). 2 randomised, crossover, double-blind, passive placebo-controlled trial, 1 open-label trial.  Ketamine dosage: IV at 0.5 mg/kg over 40mins.  Number of doses: 1 or on patient-by-patient basis. | Significant and rapid reductions in depressive symptoms – observed as early as 40 minutes post-infusion.  Response rates observed in as many as 79% of patients. |
| Katalinic et al., 2013  Australia & NZ  Systematic review. | Studies published in the English language and peer-reviewed journals, incorporating depressed patients administered ketamine and assessing mood outcomes. | Summary measures:  Montgomery-Asberg, Hamilton Rating Scale, Becks Depression Inventory,  Synthesis:  Narrative synthesis | 3 studies assessed BD patients. (N range = 2 – 18). 2 randomised, crossover, double-blind, passive placebo-controlled trial and N = 1 case study.  Ketamine dosage range: single IV infusion at 0.5 mg/kg over 40mins or IM at 32 – 50mg every 3 days.  Oral and intranansal also trialled in case study, although no data reported. | RCTs: Diazgranados et al. & Zarate et al. observed clinical improvement in 71% and 79% of patients respectively, maintained for 14 days post-infusion/s  Case study: Cusin et al 2012:  complete remission following 32mg infusion of IM ketamine in patient 1 and reduced symptoms after 1 week in patient 2. Partial relapses observed at 5 and 6 months respectively. |
| Caddy et al., 2014  United Kingdom.  Systematic review and meta-analysis | Studies that assessed adult populations (≥ 18yrs) diagnosed with MDD or BPAD in accordance with structured diagnostic interview.  Studies which assessed the impact of ketamine on depressed mood, mood symptoms and/or suicidality. | Summary measures:  Hamilton Rating Scale for Depression, Montgomery-Asberg, Hamilton Rating Scale or Becks Depression Inventory.  Synthesis:  Narrative synthesis and meta-analysis (Forest Plot) | 2 studies assessed BD and 1 assessed MDD and BPAD together. (N = 33)  Both randomised, crossover, double-blind, passive placebo-controlled trials.  Ketamine dosage: 0.5 mg/kg IV ketamine over 40mins or Saline placebo. Single dose | Reduction in depressive symptoms observed as early as 40 mins following infusion, relative to placebo – sustained to Day 3 across both studies. (P = < 0.001)  Ketamine vs placebo 60-80mins post-infusion: −3.406 (p < 0.001; 95% CI −6.303 to −0.509)  210-230mins post-infusion: −5.371 (p < 0.001; 95% CI −6.574 to −4.168).  Comparable response (71% - 79%) and remission (29-31%) rates. |
| Coyle & Laws., 2015  United Kingdom.  Systematic review and meta-analysis | Studies which involved administration of at least one ketamine infusion as treatment for depression and comprised of at least eight patients.  Patients’ primary diagnosis could be either MDD or BD.  Studies which recorded depressive symptoms in accordance with standardised assessment scale (eg. DSM-IV) | Summary measures:  Montgomery-Asberg, Hamilton Rating Scale, Becks Depression Inventory  Synthesis:  Narrative synthesis and random effect model for meta-analysis. | 3 studies reported on BD. (N total = 59)  N = 2 RTCs with cross-over design and 1 open-label trial.  Ketamine dosage: IV at 0.5 mg/kg over 40mins or Saline placebo. Single dose | Pooled effect sizes indicate ketamine treatment consistently reduced depressive symptoms across all assessed timepoints.  Peak response time for BD samples suggested to be 7 days.  For BD, effect sizes ranged from moderate at 24 h (- 0.64, C.I. = - 0.79 - - 0.49, P = < 0.001)  to (- 1.51, C.I. = - 1.99 = - 1.03, P = < 0.001) at 7 days post-infusion. |
| McGirr et al., 2015  Canada & USA.  Systematic review and meta-analysis | Studies published in the English language, where patients were randomised to conditions, double-blind, placebo controlled and where the design was cross-over or parallel.  Sample sample-size greater than 5 patients, adults diagnosed with major depressive episode (unipolar or bipolar) in accordance with standardised assessment scale. Ketamine treatment of single administration (oral, intranasal or parenteral route.) | Summary measures:  Hamilton Rating Scale for Depression or Montgomery-Asberg.  Synthesis:  Narrative synthesis and random effects model for meta-analysis. | 3 studies assessed patients with BD. (N total =34)  All randomised, crossover, double-blind, passive placebo-controlled trials.  Ketamine dosage: 0.5 mg/kg IV ketamine over 40mins or Saline placebo. Single dose | In the ketamine group, 22% - 28%  were remitted on Day 1, 7% - 22% on Day 3 and 11% - 28% on Day 7, relative to 0% of placebo group.  After 24 h: Pooled OR = 7.06 (95% C.I. = 2.50– 19.95, P = < 0.001) for clinical remission and 9.10 (95% C.I. = 4.28–19.34, P = <0.001) for clinical response.  After 7 days, Pooled OR was 4.00 (95% CI 1.52– 10.51, z= 2.81, p< 0.01) for clinical remission and 4.87 (95% CI 2.24–10.55, z= 4.01, p< 0.001) for clinical response.  Depression scores for BD specific studies: Overall SMD: 0.68 (0.51 – 0.86) P = 0.000 |
| Fond et al., 2014  France.  Systematic review and meta-analysis | Studies of a randomised controlled trial nature, with at least one administration of ketamine in isolation or with a second anaesthetic agent.  Patients diagnosed with major depression or bipolar, in accordance with validated scales (eg. DSM-IV), treatment- resistant or not. | Summary measures:  Montgomery-Asberg or Hamilton Rating Scale for Depression.  Synthesis:  Narrative synthesis. Fixed and random effects models for meta-analysis. | 3 studies assessed BD (N total = 34)  All randomised, crossover, double-blind, passive placebo-controlled trials.  Ketamine dosage: 0.5 mg/kg IV ketamine over 40mins or Saline placebo. Single dose. | Ketamine elicits rapid improvement in depressive symptoms relative to placebo.  Within 24hours post-infusion, depression scores were significantly reduced in the BD ketamine patients, relative to controls. (SMD =−1.34; 95 % C.I. = −1.94, −0.75; P = <0.01) |
| Romeo et al., 2015  France.  Systematic review and meta-analysis | Studies published in the English language, in peer-reviewed journals.  Randomized, double-blind and placebo-controlled trials of ketamine, including patients diagnosed with major depressive episode in accordance with validated scales (eg. DSM, III, IV or V) | Summary measures:  Montgomery-Asberg or Hamilton Rating Scale for Depression.  Synthesis:  Narrative synthesis. and random effects model for meta-analysis. | 3 studies assessed BD (N total = 34). All randomised, crossover, double-blind, passive placebo-controlled trials.  Ketamine dosage: 0.5 mg/kg of IV ketamine over 40mins or Saline placebo. Single dose. | Ketamine contributed to rapid reduction in symptoms for BD samples, relative to placebo – however, efficacy failed to reach significance after 4 days.  SMDs overall effect: SMD = 0.06, 95% C.I. = 0.22 - 0.33, P = 0.67  Mean percentages of improvement, weighted for sample size, from baseline depression scores showed a major efficacy of ketamine group on Day 1 (-41.17% vs - 6.06%), through to Day 7 (- 20.04% vs - 7.18%) and Day 14 (- 15.38% vs -5.87%) |
| Kishimoto et al., 2016  Japan & USA  Meta-analysis | Studies employing a parallel-group or cross-over randomised controlled-trail design.  Studies which assessed a single IV administration of ketamine against a placebo/pseudo-placebo on patients with MDD or BD. | Summary measures:  Hamilton Rating Scale for Depression or Montgomery-Asberg.  Synthesis:  Random-effects model. | 3 studies assessed BD (N total = 34). All randomised, crossover, double-blind, placebo-controlled trials.  Ketamine dosage: 0.1 mg/kg – 0.5 mg/kg or Saline placebo. Single dose. | Pooled data indicates a robust and heightened effect of ketamine relative to placebo, in reducing depressive symptoms, within 40-60mins following infusion: Hedges’ g = −0.50 (95% C.I. = 1.00 to −0.00, P = 0.05) which extended as far as 8 days: Hedges’ g = −0.38 (95% C.I. = −0.73 to −0.03, P = 0.036)  Superior and significant response and remission rates with ketamine up to 7 days for Response: RR = 3.43, (95% C.I. = 1.77–6.63, P < 0.001) and 5 days post-infusion for Remission: RR = 5.22, (95% C.I. = 1.20–22.6, P = 0.03) |
| Rosenblat et al., 2019  Canada and Australia  Systematic Review | Human studies published in the English language, examining the antidepressant effects of oral/sublingual racemic, S-ketamine or R-ketamine.  No limitations in relation to study quality (eg. randomisation or blinding protocols | Summary measures:  Hamilton Rating Scale for Depression, Montgomery-Asberg or Clinical Global Impression Scale.  Primary focus of review was on rapid (within initial 24hours antidepressant effects)  Synthesis:  Narrative synthesis | 2 studies assessed BD patients. (N total = 28)  1 chart review and 1 case study.  Ketamine dosage: 10mg (sublingual) or 0.5 mg/kg – 3 mg/kg  Number of doses not specified. | Therapeutic effects of ketamine observed as soon as 90minutes following infusion in the chart review.  The case study reported  reductions in depressive symptoms observed within initial 24hours for both patients and maintained clinical responses observed. |
| McIntyre et al., 2020  Canada, USA & Singapore  Systematic review and meta-analysis | Studies on humans diagnosed with depression via DMS.  Studies employing a randomised, double-blind placebo-controlled design, providing data at the specific time-points: 24 h, 2–6 days, 7–20 days, 21–28 days | Summary measures:  Montgomery-Asberg or Hamilton Rating Scale for Depression  Synthesis:  Random-effects model. | 2 studies assessed patients with BD. Both randomised, crossover, double-blind, passive placebo-controlled trials.  Ketamine dosage: IV at 0.5 mg/kg over 40mins or Saline placebo. Single dose. | Large and significant effects of ketamine treatment across methods of administration and across all time-points, as soon as 24 hours post-treatment.  Pooled effect size for studies including BD patients: g = 0.406 (N = 20, 95% C.I. = 0.261 to 0.552, P = < 0.01). |
