# Supplementary material for: Ketamine for the treatment of mental health and substance use disorders: comprehensive systematic review
Source: BJPsych Open. 2021 Dec 23;8(1):e19. doi: 10.1192/bjo.2021.1061 (PMC8715255; doi:10.1192/bjo.2021.1061)
Supplement: Supplementary file 1 [file S2056472421010619sup001.zip › S2056472421010619sup010.docx]

| **Table 2:** Characteristics and results of included studies on depression | | | | |
| --- | --- | --- | --- | --- |
| **Authors, date, location & design:** | **Eligibility criteria:** | **Summary measure & Synthesis of results:** | **Study characteristics:** | **Results:** |
| Aan Het Rot et al, 2012  Netherlands & USA.  Systematic review | Studies including patients who met clinical criteria for a mood disorder, were in receipt of ketamine to study its antidepressant effects and were assessed clinically for at least 230 min (~4 hours) post-ketamine treatment. | Summary measure:  Becks Depression Inventory, Hamilton Rating Scale and Montgomery-Asberg  Synthesis:  Narrative synthesis | 21 studies reviewed (N = 163): 8 case studies, 10 open label & 3 controlled trials.  Patients typically diagnosed with comorbid current or lifetime anxiety.  Ketamine dosage range: 0.27 mg/kg - 1mg/kg over 40-60mins. Number of infusions ranges from 1-6. | Rapid antidepressant effects of ketamine observed from early as 4 hours (13% - 100%), at 24 hours (25% - 85%) and at 72 hours (14% - 70%) |
| Caddy et al., 2014  United Kingdom.  Systematic review and meta-analysis | Studies that assessed adult populations (≥ 18yrs) diagnosed with MDD or BPAD in accordance with structured diagnostic interview.  Studies which assessed the impact of ketamine on depressed mood, mood symptoms and/or suicidality. | Summary measures:  Montgomery-Asberg, Hamilton Rating Scale, Becks Depression Inventory & HARS.  Synthesis:  Narrative synthesis and meta-analysis | 22 Randomised controlled trials. N = 629 participants.  Ketamine dosage range: 0.2 mg/kg - 0.5 mg/kg, typically over 40mins. Mostly single infusions however some multiple infusions (3-11).  5 assessed in ECT context. | Mean differences in HDRS score between ketamine and placebo reported at: 60-80mins: −3.406 (p < 0.001; 95% CI −6.303 to −0.509) and 210-230mins: −5.371 (p < 0.001; 95% CI −6.574 to −4.168  Response rate with ketamine ranged from 43% - 92.3% & and Remission from 26% - 50%. |
| Caddy et al., 2015  United Kingdom, Israel & Japan.  Systematic review and meta-analysis | Included studies with a double-blind or single-blind RCT design. At least 80% of participants had primary diagnosis of MDD. | Summary measures  Hamilton Rating Scale or Montgomery-Asberg.  Synthesis  Narrative synthesis and random effects | 25 studies included (N = 1242 participants)  5 studies in meta-analysis. All randomised, double-blind controlled trials.  Ketamine dosage range: IV from 0.4 mg/kg-0.5mg/kg,  1 to 3 infusions. | 24hours: Ketamine relative to placebo SMD = - 1.42 (95% C.I. = - 2.26 to - 0.57, P = 0.001) and 1 week: SMD = - 0.62 (95% C.I. = - 1.00 to- 0.24, P = 0.001), at 2 weeks. SMD = - 0.10 (95% C.I. =  - 0.68 – 0.48, P = 0.74) |
| Covvey et al., 2012  USA.  Systematic review | Studies published in the English-language, evaluating ketamine’s effect on depressive symptoms in humans with treatment-resistant MDD | Summary measures  Hamilton Rating Scale for Depression, Becks Depression Inventory or Montgomery-Asberg  Synthesis  Narrative synthesis | 3 case studies, 1 case report, 3 open-label 1 randomised cross-over trial included  Ketamine dosage range: 0.15 mg/kg – 0.5 mg/kg. 1-6 doses. | Case reports: Effect onset emerged from as early as 1-2 days and lasted between several days to 3 months in one study.  Clinical trials:  Treatment response observed as early as 2 hours post-infusion and extended up to 7-days later. Remission achieved as early as within 1-day post-treatment. |
| Coyle & Laws 2015  United Kingdom.  Systematic review and meta-analysis | Studies which involved administration of at least one ketamine infusion as treatment for depression and comprised of at least eight patients.  Studies which recorded depressive symptoms in accordance with standardised assessment scale (eg. DSM-IV) | Summary measures:  Montgomery-Asberg, Hamilton Rating Scale, Becks Depression Inventory & HARS.  Synthesis:  Narrative synthesis and random effect model for meta-analysis. | 21 studies were reviewed (N = 437), study designs not specified.  N = 17 studies followed a single-infusion protocol, number of doses in repeat administration studies not specified.  Ketamine dosage range: unspecified. | Robust effect of ketamine treatment in reduction of depressive symptoms. Large and significant Hedge’s g values across all time-points. At 4 hours: - 1.29 (95% C.I. = - 1.66 to – 0.92, P = <0.001)  At 7 days: - 1.06 (95% C.I. = - 1.57 to – 0.55, P = <0.001)  And 12-14 days: - 1.67 (95% C.I. = - 2.85 to – 0.49, P = 0.006) |
| Fond et al., 2014  France.  Systematic review and meta-analysis | Studies of a randomised controlled trial nature, with at least one administration of ketamine in isolation or with a second anaesthetic agent.  Patients diagnosed with major depression or bipolar, in accordance with validated scales (eg. DSM-IV), treatment- resistant or not. | Summary measures:  Hamilton Rating Scale for Depression or Montgomery-Asberg.  Synthesis:  Narrative synthesis. Fixed and random effects models for meta-analysis. | 9 non-ECT and 4 ECT studies (N = 192 with MDD) combination of cases and controls.  All randomised controlled trials.  N = 163 cases administered ketamine hydrochloride and N = 148 controls administered placebo or midazolam.  Ketamine dosage range: 0.4 mg/kg – 0.8 mg/kg. Number of doses not specified. | Significant decreases in depression symptoms relative to placebo in non- ECT context: SMD = - 0.99 (95% C.I. - -1.23 to – 0.75, P = < 0.001) and ECT-context: SMD=−0.56; 95 % CI −1.10, −0.02; p=0.04 which can be sustained for up to 3 days. |
| Garay et al., 2017  France.  Systematic review | Agents assessed were required to be tested in phase II and/or phase III clinical trials. | Summary measures:  Montgomery-Asberg or Hamilton Rating Scale for Depression.  Synthesis:  Narrative synthesis | IV ketamine:  9 phase II or III clinical trials (N range = 20-100), Ketamine dosage range: 0.2 mg/kg - 0.5 mg/kg  IN esketamine:  7 phase II or III clinical trials (N range = 30-1071). Ketamine dosage range: 0.20 mg/kg – 0.40 mg/kg, 28 mg – 48mg.  Number of doses not specified. | Phase II clinical trials highlight rapid and efficacious impact of IV ketamine treatment, extending up to 15 days post-infusion.  Efficacy of IN (esketamine) relative to placebo, observed within 40 minutes following infusion and sustained up to 8 days later. |
| Han et al., 2016  China.  Meta-analysis. | Studied required to employ a randomised, double-blind placebo-controlled protocol (placebo active or inactive)  Individuals diagnosed with MDD. | Summary measures:  Hamilton Rating Scale for Depression or Montgomery-Asberg (response rate as primary outcome)  Synthesis:  Random-effects model. | 9 studies reviewed (N = 368 patients)  All randomised, double-blind controlled trials.  Ketamine dosage range: 0.5 mg/kg – 0.54 mg/kg. Single dose | Response with ketamine vs placebo at 24 hours: Pooled OR = 10.09 (95% C.I: 4.96–20.52, P = <0.00001) and Day 7: OR = 5.66 (95% CI: 2.92–10.97, P = < 0.00001)  Remission with ketamine vs placebo at 24 hours: Pooled OR = 5.25 (95% C.I: 1.82–15.17, P = 0.002) and Day 7 = OR = 4.60 (95% CI: 1.88–11.26, P = 0.0008) |
| Hasselmann., 2014  The Netherlands.  Systematic review. | Duration of clinical assessment required to be >30min following treatment.  Patients to have received formal diagnosis of mood disorder and studies limited to humans. | Summary measures:  Hamilton Rating Scale for Depression.  Synthesis:  Narrative synthesis | 28 studies reviewed**.** 21 Open label trials, 6 randomised controlled trials.  Typical ketamine dosage: 0.5 mg/kg over 40mins. Ketamine infusions ranged from 1 – 8 (majority single) | Clinical response observed as early as 40 minutes and in up to 70% of one sample.  Depressive symptoms seen to reduce by as much as 85% |
| Jankauskas et al., 2018  Canada & USA.  Systematic Review | Studies published in the English language, with a randomised controlled-trial design with MDD samples.  Studies excluded where ketamine was administered in a non-ECT setting at dose of 0.5 mg/kg.  Studies with a cross-over protocol also excluded. | Summary measures:  Montgomery-Asberg or Hamilton Rating Scale for Depression  Synthesis:  Narrative synthesis | 26 studies reviewed. Studies included which assessed ketamine alone without additional ECT, ketamine with additional agents without ECT and ketamine as an adjunct to ECT alone or ketamine in conjunction with other agents for ECT (eg. propofol)  All randomised controlled trials.  Ketamine dosage range: 0.3 mg/kg – 2.0 mg/kg / 28mg - 84mg. Number of doses not specified. | ECT context  Just over 40% of studies identified significantly elevated improvements in depressive symptoms following ECT, with ketamine alone or ketamine + propofol, relative to propofol alone.  Non-ECT context  Significant improvement with ketamine observed as early as 24hours. |
| Katalinic et al., 2013  Australia and NZ.  Systematic review | Studies published in the English language and peer-reviewed journals, incorporating depressed patients administered ketamine and assessing mood outcomes. All study designs were considered. | Summary measures:  Montgomery-Asberg, Hamilton Rating Scale, Becks Depression Inventory,  Synthesis:  Qualitative and Narrative synthesis | 18 studies which assessed TRD or MDD. 5 randomised controlled trials, 12 open label and 1 intraindividual cross-over.  Ketamine dosage: all studies reviewed administered ≤1 mg/kg of IV ketamine. | Broadly rapid and significant effects of ketamine treatment observed as soon as 40 mins post-infusion. |
| Kishimoto et al., 2016  Japan & USA.  Meta-analysis | Studies employing a parallel-group or cross-over randomised controlled-trail design.  Studies which assessed a single IV administration of ketamine against a placebo/pseudo-placebo on patients with MDD.  Studies which assessed oral or intranasal administration were excluded. | Summary measures:  Hamilton Rating Scale for Depression or Montgomery-Asberg.  Synthesis:  Random-effects model. | 9 ketamine studies included (N = 234 patients; MDD and BD). All nine studies were randomised controlled trials.  Ketamine dosage range: 0.1 mg/kg – 0.5 mg/kg. Single dose | Significantly greater response rates with ketamine from 40 minutes: RR = 13.6, (95% C.I. = 2.67–69.6, P = 0.00)  up to 7 days: RR = 3.43, (95% C.I. = 1.77–6.63, P < 0.001).Significantly greater remission rates from 80 minutes up to 3-5 days post-infusion: RR = 6.63, (95% C.I. = 1.23–35.7, P = 0.03) up to 5 days: RR = 5.22, (95% C.I. = 1.20–22.6, P = 0.03) |
| Kleeblatt et al, 2017  Germany.  Systematic review | Studies published in the English language – particular focus on meta-analyses, randomised controlled trials, non-randomised comparative studies and case studies.  Excluded studies administering drug in a monotherapeutic context. | Summary measures:  Montgomery-Asberg.  Synthesis:  Narrative synthesis | 2 studies assessed ketamine treatment. (N = 97 patients) - both double-blind RCTs  Ketamine dosage: 0.5 mg/kg assessed against placebo. Between 1 - 12 doses. | Ketamine highly efficacious and rapid in single-infusion context.  Response and Remission rates up to 92.3% and 76.9% respectively reported vs 51.7% and 14.3% respectively in placebo group.  Extent of long-term effects to be ascertained. |
| Kolar., 2018  Canada.  Systematic review | Studies employing a placebo-controlled randomised protocol, non-randomised controlled studies, meta-analyses, naturalistic studies, case reports, and treatment guidelines published in the past 10 years.  Studies with patients diagnosed in accordance with DSM-5. | Summary measures:  Hamilton Rating Scale for Depression or Montgomery-Asberg  Synthesis:  Narrative synthesis | 15 studies assessed ketamine treatment.  One meta-analysis including 7 RCT’s, individual randomised controlled trials and case reports  Ketamine dosage & number of doses: unspecified. | Rapid impact of ketamine treatment on depressive symptoms, observed as early as 2-4 hours post-infusion, although typically only maintained for up to 7 days, with as many as 55% of patients no longer indicating treatment response by day 7, in one study. |
| Kraus et al., 2017  Austria.  Systematic review | Clinical trials, published in a peer reviewed journal, unipolar or bipolar depression using DSM-IV or similar criteria, only standardized or reliable measures used. | Summary measure:  Hamilton Rating Scale for Depression and/or Montgomery-Asberg or Becks Depression Inventory.  Synthesis:  Narrative synthesis | 12 studies included. 7 double-blind, crossover RCTs, 5 open-label trials, (N = 226 MDD patients)  Ketamine dosage range across studies: 0.2 mg/kg – 0.5 mg/kg. Between 1-6 doses. | IV and IN ketamine are highly effective and rapid acting – producing Response rates of up to 88% at 24 hours post-infusion - greatest effects emerged at 24 hours post-infusion.  Average score reduction on Hamilton Depression Rating Scale = 10.9 and on Montgomery-Asberg Depression Rating Scale = 20.8  However, relapse rates up to 92% recorded by 2 weeks. |
| Lee et al., 2015  USA.  Systematic review and meta-analysis | Placebo-controlled double-blind studies published in the English language. Randomised, controlled trials of ketamine on patients with MDD or BP.  Studied including adult out-patients, diagnosed with standardized scales | Summary measure:  Becks Depression Inventory or Montgomery-Asberg.)  Synthesis: Narrative synthesis and Random effects model meta-analysis. | 6 randomised, placebo-controlled studies reviewed. 3 studies of exclusively MDD.  Ketamine dosage of 0.27- 0.5 mg/kg administered over 20 to 40mins. 5 single dose studies and 1 study involved a loading dose procedure (one over 10 mins and a second over 20mins, both 0.27 mg/kg) | Effects of ketamine on depressive symptoms robust and rapid. 4 studies observed between 64-71% response rate.)  Large effect size obtained for Day 1 post-infusion: SMD = 1.01; 95% C.I. = 0.69-1.34; P = <.001). Anti-depressant effect maintained at 7 days post-infusion: SMD = 0.41; 95% C.I. = 0.14-0.68; P = .003) |
| McGirr et al., 2014  Canada & USA.  Systematic review and meta-analysis | Randomised placebo controlled double blind (parallel or cross over) studies of a single dose of ketamine  published in the English language. Sample sample-size greater than 5 patients, adults diagnosed with major depressive episode (unipolar or bipolar) in accordance with standardised scales. | Summary measures:  Montgomery-Asberg or Hamilton Rating Scale for Depression  Synthesis:  Narrative synthesis and random effects model for meta-analysis. | 7 RCTs reviewed (6 exclusively MDD). N = 183 in total (N = 149 exclusively MDD)  6 studies of IV ketamine (dosage typically 0.5 mg/kg over 40mins). 1 IN ketamine (dosage 0.27 mg/kg + 0.27 mg/kg over 20mins). All single dose except 1 study which involved a loading dose procedure (one over 10 mins and a second over 20mins, both 0.27 mg/kg) | Increased response and remission rates following ketamine treatments, compared to placebo at 24 hours (pooled OR for Remission: 7.06 (95% C.I. = 2.50 – 19.95, P = < 0.001) and pooled OR for Response: 9.10 (95% C.I. = 4.28 –19.34, P = < 0.001)  And 7 days (Pooled OR for Remission: 4.00 (95% C.I. = 1.52 – 10.51, P = < 0.01) and pooled OR for Response: 4.87 (95% CI 2.24–10.55, P = < 0.001)) |
| Papadimitropoulou et al., 2017  Netherlands, UK & Germany.  Systematic review and meta-analysis. | Studies published in the English language  Adults diagnosed with treatment resistant MDD.  RCTs including < 10 patients were excluded. | Summary measures:  Montgomery-Asberg or Hamilton Rating Scale for Depression.  Synthesis:  Narrative synthesis and random-effects model for meta-analysis. | 5 studies reviewed ketamine treatment specifically. Open label or Double blind, randomised, placebo-controlled trials.  Ketamine dose or number of doses not specified. | Rapid and robust antidepressant effect of ketamine relative to placebo. Faster reductions in symptom severity and x14 greater response rate at 2 weeks.    Mean differences in change from baseline for MADRS scores as primary outcome):  -14 (95% C.I. = -19.9 to -8.0) and this change was significant. |
| Serafini et al., 2014  Italy & USA.  Systematic review. | Studies published in the English language and in a peer-reviewed journal, specifically assessing the effect of ketamine in context of TRD. | Summary measures:  Montgomery-Asberg or Young Mania Rating Scale (where specified or Becks Depression Inventory)  Synthesis:  Narrative synthesis | 22 studies were reviewed (N = 416 patients). 8 case reports/series, 5 open-label studies, 3 randomised, double-blind placebo or active-controlled studies and 6 investigating neurobiological effects/mechanisms of ketamine (all open label trials)  Ketamine dosage: 0.25 – 1.25 mg/kg. 1 - 14doses. | Rapid antidepressant effect of ketamine treatment observed at earliest, within 2 hours following administration. |
| Romeo et al., 2015  France.  Systematic review and meta-analysis | Studies published in the English language, in peer-reviewed journals.  Randomised, double-blind and placebo-controlled trials of ketamine, including patients diagnosed with major depressive episode in accordance with validated scales (eg. DSM, III, IV or V) | Summary measures:  Hamilton Rating Scale for Depression, Montgomery-Asberg.  Synthesis:  Narrative synthesis. and random effects model for meta-analysis. | 6 studies reviewed.  6 studies of IV ketamine (dosage typically 0.5 mg/kg over 40mins). 1 IN ketamine (dosage 0.27 mg/kg + 0.27 mg/kg over 20mins)  Ketamine dosage range (**IV**): 0.27 mg/kg – 0.5 mg/kg  Ketamine dosage (**IN**): 50mg.  5 single dose studies and 1 study involved a loading dose procedure (one over 10 mins and a second over 20mins, both 0.27 mg/kg) | Significant antidepressant effect of ketamine observed from Day 1 - Day 7 post-infusion, relative to placebo. Lack of sustained effects beyond 14 days.  Overall effect Day 1: SMD = - 1 (95% C.I. = -1.3 to -0.71, P = < - 0.00001) at Day 7: SMD: - 0.36 (95% C.I. – 0.65 to  - 0.08, P = 0.01) and at Day 14:  SMD = -0.38 (95% C.I. = -0.87 to 0.11, P = 0.13) |
| Rosenblat et al., 2019  Canada & Australia.  Systematic review. | Human studies published in the English language, examining the antidepressant effects of oral/sublingual racemic, S-ketamine or R-ketamine. | Summary measures:  Hamilton Rating Scale for Depression, Montgomery-Asberg or Clinical Global Impression Scale.  Synthesis:  Narrative synthesis | 13 studies reviewed. 2 proof-of-concept RCTs, 1 open-label study, 5 retrospective chart reviews and 5 case reports.  Included patients with comorbid diagnoses of PTSD and BD.  Ketamine dosage range: IV ketamine: 0.25 mg/kg – 05 mg/kg, Oral ketamine: 7.0 mg/kg - 150mg and Sublingual: 0.5 – 10mg  Number of doses:  Varies markedly (eg. from 1 dose every day for 28 days, to every 2-3 days weekly for up to 6 months. | Significant improvements in depressive symptoms observed as early as within first 90minutes post-infusion, however in certain cases (oral ketamine) this significant effect relative to control, only emerges following between 2 and 6 -weeks post-treatment. |
| McIntyre et al., 2020  Canada, USA & Singapore.  Systematic review and meta-analysis. | Studies on humans diagnosed with depression via DMS.  Studies employing a randomised, double-blind placebo-controlled design, providing data at the specific time-points: 24 h, 2–6 days, 7–20 days, 21–28 days | Summary measures:  Montgomery-Asberg or Hamilton Rating Scale for Depression  Synthesis:  Random-effects model. | 21 studies reviewed.  N = 14 IV ketamine, N =4 IN ketamine and N = 3 oral ketamine  Ketamine dosage range (IV): 0.1 mg/kg – 1 mg/kg.  1 – 4 doses. | Large and significant effects of ketamine treatment across methods of administration and across all time-points, as soon as 24 hours post-treatment.  Pooled effect size across administration methods:  g = 0.529 (95% C.I. = 0.328 – 0.729, P = < 0.01) |
| Zheng et al., 2020  China, Singapore, Australia & Macau.  Systematic review. | Studies assessing adults with MDD – treatment refractory indicators and/or suicidal ideation.  Studies comparing anti-depressant with adjunctive IN esketamine, against anti-depressants combined with IN placebo. | Summary measures:  Montgomery-Asberg Depression Rating Scale or Hamilton Rating Scale for Depression.  Synthesis:  Narrative synthesis | 4 Randomised controlled trials (n = 707 patients, 419 received IN ketamine)  Ketamine dosage range: 28mg – 84mg per administration (either x2 or x8 doses) | Significant effect of ketamine emerges as early as within initial 2hours post-infusion, showing 50% response rate and 23.5% remission rate, relative to 18.2% response and 3.0% remission in the control group.  Effect sustained for at least 28 days, with response rate of 58.7% and remission at 42.3% with ketamine relative to 43.0% response and 30.7% remission in the control group. |
| Papakostas et al., 2020  USA.  Meta-analysis. | Studies employing a randomised, double-blind design – assessing adjunctive intranasal esketamine against a placebo on patients diagnosed with MDD exclusively. | Summary measures:  Montgomery-Asberg  Synthesis:  Random-effects model. | 5 randomised double-blind controlled trials were reviewed (N = 774).  Ketamine dosage range: 28mg – 84mg.  Number of doses: 4 - 8  All studies included ketamine as an adjunct to existing antidepressants. | 40% - 45% increased chance of response and remission following IN esketamine, relative to placebo.  MADRS score change (primary outcome):  Overall SMD = 0.36 (95%, C.I. = 0.24-0.49. P = < 0.0001) |
