# Supplementary material for: Ketamine for the treatment of mental health and substance use disorders: comprehensive systematic review
Source: BJPsych Open. 2021 Dec 23;8(1):e19. doi: 10.1192/bjo.2021.1061 (PMC8715255; doi:10.1192/bjo.2021.1061)
Supplement: Supplementary file 1 [file S2056472421010619sup001.zip › S2056472421010619sup011.docx]

**Supplementary Materials:**

**Table 1:** Studies which may appear to meet inclusion criteria but were excluded and reasons for exclusion

| Reference: | Exclusion reason |
| --- | --- |
| Krystal, J. H., Madonick, S., Perry, E., Gueorguieva, R., Brush, L., Wray, Y., Belger, A., & D'Souza, D. C. (2006). Potentiation of low dose ketamine effects by naltrexone: potential implications for the pharmacotherapy of alcoholism. *Neuropsychopharmacology*, *31*(8), 1793–1800 | This study does not focus primarily the effects of ketamine but on the interplay of ketamine and naltrexone |
| Nielsen, R. V., Fomsgaard, J. S., Nikolajsen, L., Dahl, J. B., & Mathiesen, O. (2019). Intraoperative S-ketamine for the reduction of opioid consumption and pain one year after spine surgery: A randomized clinical trial of opioid-dependent patients. *European Journal of Pain, 23*(3), 455-460. | The definition of opioid dependence in this study based solely on 6 months use of opioids does not meet the diagnostic criteria for opioid use disorders. |
| Das, R.K., Gale, G., Walsh, K. et al. Ketamine can reduce harmful drinking by pharmacologically rewriting drinking memories. *Nat Commun.* *10,* 5187, 1-10 | Participants in this study are harmful drinkers, not those with alcohol use disorders. |
| Rodriguez, C. I., Wheaton, M., Zwerling, J., Steinman, S. A., Sonnenfeld, D., Galfalvy, H., & Simpson, H. B. (2016). Can exposure-based CBT extend the effects of intravenous ketamine in obsessive-compulsive disorder? an open-label trial. *The Journal of clinical psychiatry, 77*(3), 408–409. | This focuses on whether CBT can extend the effects of ketamine not specifically on the therapeutic effects of ketamine. |
| Sappington, A.A., Corssen, G., Becker, A.T. and Tavakoli, M. (1979). Ketamine ‐ facilitated induced anxiety therapy and its effect upon clients' reactions to stressful situations. *J. Clin. Psychol., 35*, 425-429. | Ketamine is used in this study in order to facilitate induced anxiety therapy. |
| Hartberg, J., Garrett-Walcott, S., & De Gioannis, A. (2018). Impact of oral ketamine augmentation on hospital admissions in treatment-resistant depression and PTSD: a retrospective study. *Psychopharmacology, 235*(2), 393–398. | This study does not report on PTSD symptom outcomes necessary for the synthesis |
| Pradhan, B., Mitrev, L., Moaddell, R., & Wainer, I. W. (2018). d-Serine is a potential biomarker for clinical response in treatment of post-traumatic stress disorder using (R,S)-ketamine infusion and TIMBER psychotherapy: A pilot study. *Biochimica et biophysica acta. Proteins and proteomics, 1866*(7), 831-839. | This is primarily focused on predictors of treatment response. |
| Kronish, I. M., et al. (2018). "Personalized (N-of-1) Trials for Depression- A Systematic Review." *Journal of clinical psychopharmacology, 38*(3)- 218-225 | Ketamine is not the primary focus of this systematic review and only one ketamine study was included. |
| Schoevers, R., Chaves, T., Balukova, S., Rot, M., & Kortekaas, R. (2016). Oral ketamine for the treatment of pain and treatment-resistant depression. *British Journal of Psychiatry, 208*(2), 108-113. doi:10.1192/bjp.bp.115.165498 | This review examines ketamine for depression in the context of chronic pain, which is not the focus of the current review. |
| Peyrovian, B., McIntyre, R. S., Phan, L., Lui, L. M. W., Gill,  H., Majeed, A., . . . Rosenblat, J. D. (2020). Registered clinical trials investigating ketamine for psychiatric disorders*. Journal of psychiatric research, 127*, 1-12. | This is a review of registered trials of ketamine’s therapeutic effects, rather than published trials. |
| Cao, B., Zhu, J., Zuckerman, H., Rosenblat, J. D., Brietzke, E., Pan, Z., . . . McIntyre, R. S. (2019). Pharmacological interventions targeting anhedonia in patients with major depressive disorder: A systematic review. *Progress in neuro-psychopharmacology & biological psychiatry, 92*, 109-117. | This review focuses on a single symptom of depression which is not the focus of the current systematic review. |
| Erdil, F., Ozgul, U., Çolak, C., Cumurcu, B., & Durmus, M. (2015). Effect of the Addition of Ketamine to Sevoflurane Anesthesia on Seizure Duration in Electroconvulsive Therapy. *The journal of ECT, 31*(3), 182–185. | Individual effects of ketamine are not examined, ketamine is combined with another anaesthetic agent. |
| Brewer, C., Davidson, J., & Hereward, S. (1972). Ketamine (“Ketalar”): A Safer Anaesthetic for ECT. *British Journal of Psychiatry*, *120*(559), 679-680. | This study does not report necessary data for extraction and synthesis. |
| Abdallah, C. G., Fasula, M., Kelmendi, B., Sanacora, G., & Ostroff, R. (2012). Rapid antidepressant effect of ketamine in the electroconvulsive therapy setting. The journal of ECT, 28(3), 157–161. | Individual effects of ketamine are not examined, ketamine is combined with another anaesthetic agent. |
|  |  |
| Diamond, P. R., Farmery, A. D., Atkinson, S., Haldar, J., Williams, N., Cowen, P. J., Geddes, J. R., & McShane, R. (2014). Ketamine infusions for treatment resistant depression: a series of 28 patients treated weekly or twice weekly in an ECT clinic. Journal of psychopharmacology (Oxford, England), 28(6), 536–544. | In this study, ketamine infusions were simply delivered in an ECT clinic, rather than ketamine being used as an anaesthetic agent for ECT or ketamine combination with ECT. |
| Ye, J., Lin, X., Jiang, D., Chen, M., Zhang, Y., Tian, H., . . . Zhao, Y. (2019). Adjunct ketamine treatment effects on treatment-resistant depressive symptoms in chronic treatment-resistant schizophrenia patients are short-term and disassociated from regional homogeneity changes in key brain regions-A pilot study. *Psychiatry and Clinical Psychopharmacology, 29*(4), 907-915. | This study is primarily concerned with the mechanisms of ketamine treatment which beyond the scope of the current review. |
