# Supplementary material for: Ketamine for the treatment of mental health and substance use disorders: comprehensive systematic review
Source: BJPsych Open. 2021 Dec 23;8(1):e19. doi: 10.1192/bjo.2021.1061 (PMC8715255; doi:10.1192/bjo.2021.1061)
Supplement: Supplementary file 1 [file S2056472421010619sup001.zip › S2056472421010619sup012.docx]

**PsychInfo:**

1. exp KETAMINE/

2. ketamine.mp. [mp=title, abstract, heading word, table of contents, key concepts, original title, tests & measures]

3. neuropsych*.mp. [mp=title, abstract, heading word, table of contents, key concepts, original title, tests & measures]

4. exp COGNITION/

5. cognit*.mp. [mp=title, abstract, heading word, table of contents, key concepts, original title, tests & measures]

6. exp LONG TERM MEMORY/ or exp EPISODIC MEMORY/ or exp MEMORY DISORDERS/ or exp SHORT TERM MEMORY/ or exp MEMORY/ or exp MEMORY CONSOLIDATION/ or exp SPATIAL MEMORY/

7. memory*.mp. [mp=title, abstract, heading word, table of contents, key concepts, original title, tests & measures]

8. exp "Depression (Emotion)"/ or exp Major Depression/

9. exp Affective Disorders/

10. exp DISRUPTIVE MOOD DYSREGULATION DISORDER/

11. mood*.mp. [mp=title, abstract, heading word, table of contents, key concepts, original title, tests & measures]

12. exp Bipolar Disorder/

13. bipolar*.mp. [mp=title, abstract, heading word, table of contents, key concepts, original title, tests & measures]

14. mania*.mp. [mp=title, abstract, heading word, table of contents, key concepts, original title, tests & measures]

15. exp Anxiety Disorders/

16. anxi*.mp. [mp=title, abstract, heading word, table of contents, key concepts, original title, tests & measures]

17. exp Obsessive Compulsive Disorder/

18. obsessive compulsive disorder.mp. [mp=title, abstract, heading word, table of contents, key concepts, original title, tests & measures]

19. OCD.mp. [mp=title, abstract, heading word, table of contents, key concepts, original title, tests & measures]

20. exp Generalized Anxiety Disorder/

21. generalised anxiety disorder.mp. [mp=title, abstract, heading word, table of contents, key concepts, original title, tests & measures]

22. post traumatic stress disorder.mp. [mp=title, abstract, heading word, table of contents, key concepts, original title, tests & measures]

23. exp Posttraumatic Stress Disorder/

24. ptsd.mp. [mp=title, abstract, heading word, table of contents, key concepts, original title, tests & measures]

25. exp Social Phobia/

26. social anxiety disorder.mp.

27. exp Eating Disorders/

28. eating disorder.mp. [mp=title, abstract, heading word, table of contents, key concepts, original title, tests & measures]

29. exp ANOREXIA NERVOSA/

30. anorex*.mp. [mp=title, abstract, heading word, table of contents, key concepts, original title, tests & measures]

31. exp BULIMIA/

32. bulim*.mp. [mp=title, abstract, heading word, table of contents, key concepts, original title, tests & measures]

33. exp Binge Eating Disorder/

34. binge eating disorder.mp. [mp=title, abstract, heading word, table of contents, key concepts, original title, tests & measures]

35. exp INSOMNIA/

36. insomni*.mp. [mp=title, abstract, heading word, table of contents, key concepts, original title, tests & measures]

37. exp Sleep Disorders/

38. exp Dissociative Disorders/

39. dissociat*.mp. [mp=title, abstract, heading word, table of contents, key concepts, original title, tests & measures]

40. exp Schizophrenia/ or exp Psychosis/

41. psychosis.mp. [mp=title, abstract, heading word, table of contents, key concepts, original title, tests & measures]

42. psychotic.mp. [mp=title, abstract, heading word, table of contents, key concepts, original title, tests & measures]

43. schizo*.mp. [mp=title, abstract, heading word, table of contents, key concepts, original title, tests & measures]

44. exp ATTEMPTED SUICIDE/ or exp SUICIDE/

45. exp Suicidal Ideation/

46. suicid*.mp. [mp=title, abstract, heading word, table of contents, key concepts, original title, tests & measures]

47. exp Drug Abuse/

48. exp "Substance Use Disorder"/

49. exp Drug Dependency/

50. substance dependence.mp. [mp=title, abstract, heading word, table of contents, key concepts, original title, tests & measures]

51. substance abuse.mp. [mp=title, abstract, heading word, table of contents, key concepts, original title, tests & measures]

52. exp DRUG ADDICTION/ or exp ADDICTION/

53. addiction.mp. [mp=title, abstract, heading word, table of contents, key concepts, original title, tests & measures]

54. addictive behaviour.mp.

55. "substance use disorder".mp. [mp=title, abstract, heading word, table of contents, key concepts, original title, tests & measures]

56. "drug use".mp.

57. exp Personality Disorders/

58. personality disorder.mp. [mp=title, abstract, heading word, table of contents, key concepts, original title, tests & measures]

59. exp VASCULAR DEMENTIA/ or exp DEMENTIA/ or exp SEMANTIC DEMENTIA/ or exp AIDS DEMENTIA COMPLEX/ or exp PRESENILE DEMENTIA/ or exp DEMENTIA WITH LEWY BODIES/ or exp SENILE DEMENTIA/

60. dementia.mp. [mp=title, abstract, heading word, table of contents, key concepts, original title, tests & measures]

61. exp TOBACCO SMOKING/ or exp SMOKELESS TOBACCO/

62. tobacco.mp. [mp=title, abstract, heading word, table of contents, key concepts, original title, tests & measures]

63. exp ELECTRONIC CIGARETTES/

64. cigarette.mp. [mp=title, abstract, heading word, table of contents, key concepts, original title, tests & measures]

65. exp CRACK COCAINE/ or exp COCAINE/

66. cocaine smoking.mp. [mp=title, abstract, heading word, table of contents, key concepts, original title, tests & measures]

67. pipe smoking.mp. [mp=title, abstract, heading word, table of contents, key concepts, original title, tests & measures]

68. cigar smoking.mp.

69. smoking.mp. [mp=title, abstract, heading word, table of contents, key concepts, original title, tests & measures]

70. cocaine related disorders.mp. [mp=title, abstract, heading word, table of contents, key concepts, original title, tests & measures]

71. cocaine.mp. [mp=title, abstract, heading word, table of contents, key concepts, original title, tests & measures]

72. exp ALCOHOLS/

73. alcohol.mp. [mp=title, abstract, heading word, table of contents, key concepts, original title, tests & measures]

74. exp Alcoholism/ or exp Alcohol Abuse/

75. "alcohol use disorder".mp. [mp=title, abstract, heading word, table of contents, key concepts, original title, tests & measures]

76. alcohol dependence.mp.

77. exp OPIATES/

78. OPIATE.mp. [mp=title, abstract, heading word, table of contents, key concepts, original title, tests & measures]

79. exp HEROIN ADDICTION/ or exp HEROIN/

80. HEROIN.mp. [mp=title, abstract, heading word, table of contents, key concepts, original title, tests & measures]

81. exp AMPHETAMINE/

82. amphetamine.mp. [mp=title, abstract, heading word, table of contents, key concepts, original title, tests & measures

83. exp Mental Health/

84. mental health.mp. [mp=title, abstract, heading word, table of contents, key concepts, original title, tests & measures]

85. 1 or 2

86. 3 or 4 or 5 or 6 or 7 or 8 or 9 or 10 or 11 or 12 or 13 or 14 or 15 or 16 or 17 or 18 or 19 or 20 or 21 or 22 or 23 or 24 or 25 or 26 or 27 or 28 or 29 or 30 or 31 or 32 or 33 or 34 or 35 or 36 or 37 or 38 or 39 or 40 or 41 or 42 or 43 or 44 or 45 or 46 or 47 or 48 or 49 or 50 or 51 or 52 or 53 or 54 or 55 or 56 or 57 or 58 or 59 or 60 or 61 or 62 or 63 or 64 or 65 or 66 or 67 or 68 or 69 or 70 or 71 or 72 or 73 or 74 or 75 or 76 or 77 or 78 or 79 or 80 or 81 or 82 or 83 or 84

87. 85 and 86

88. limit 87 to human

**Ovid Medline:**

1. KETAMINE/

2. ketamine.mp. [mp=title, abstract, heading word, table of contents, key concepts, original title, tests & measures]

3. neuropsych*.mp. [mp=title, abstract, heading word, table of contents, key concepts, original title, tests & measures]

4. Cognition/

5. cognit*.mp. [mp=title, abstract, heading word, table of contents, key concepts, original title, tests & measures]

6. MEMORY, SHORT-TERM/ or MEMORY/ or MEMORY, EPISODIC/ or MEMORY DISORDERS/ or SPATIAL MEMORY/ or MEMORY CONSOLIDATION/ or MEMORY, LONG-TERM/

7. memory*.mp. [mp=title, abstract, heading word, table of contents, key concepts, original title, tests & measures]

8. Depressive Disorder/ or Depression/

9. affective*.mp. [mp=title, abstract, heading word, table of contents, key concepts, original title, tests & measures]

10. Depressive Disorder, Major/ or MOOD DISORDERS/

11. mood*.mp. [mp=title, abstract, heading word, table of contents, key concepts, original title, tests & measures]

12. BIPOLAR DISORDER/

13. bipolar*.mp. [mp=title, abstract, heading word, table of contents, key concepts, original title, tests & measures]

14. mania*.mp. [mp=title, abstract, heading word, table of contents, key concepts, original title, tests & measures]

15. Anxiety Disorders/ or Anxiety/

16. anxi*.mp. [mp=title, abstract, heading word, table of contents, key concepts, original title, tests & measures]

17. Obsessive-Compulsive Disorder/

18. obsessive compulsive disorder.mp. [mp=title, abstract, heading word, table of contents, key concepts, original title, tests & measures]

19. OCD.mp. [mp=title, abstract, heading word, table of contents, key concepts, original title, tests & measures]

20. "generalised anxiety disorder".mp. [mp=title, abstract, heading word, table of contents, key concepts, original title, tests & measures]

21. Stress Disorders, Post-Traumatic/

22. post traumatic stress disorder.mp. [mp=title, abstract, heading word, table of contents, key concepts, original title, tests & measures]

23. ptsd.mp. [mp=title, abstract, heading word, table of contents, key concepts, original title, tests & measures]

24. Phobia, Social/

25. "social anxiety disorder".mp. [mp=title, abstract, heading word, table of contents, key concepts, original title, tests & measures]

26. "Feeding and Eating Disorders"/

27. eating disorder.mp. [mp=title, abstract, heading word, table of contents, key concepts, original title, tests & measures]

28. Anorexia/ or Bulimia Nervosa/ or Anorexia Nervosa/ or Bulimia/

29. anorex*.mp. [mp=title, abstract, heading word, table of contents, key concepts, original title, tests & measures]

30. Binge-Eating Disorder/

31. bulimi*.mp. [mp=title, abstract, heading word, table of contents, key concepts, original title, tests & measures]

32. insomnia.mp. [mp=title, abstract, heading word, table of contents, key concepts, original title, tests & measures]

33. "Sleep Initiation and Maintenance Disorders"/

34. Dissociative Disorders/

35. dissociat*.mp. [mp=title, abstract, heading word, table of contents, key concepts, original title, tests & measures]

36. Psychotic Disorders/

37. psychosis.mp. [mp=title, abstract, heading word, table of contents, key concepts, original title, tests & measures]

38. psychotic.mp. [mp=title, abstract, heading word, table of contents, key concepts, original title, tests & measures]

39. Schizophrenia/

40. schizo*.mp. [mp=title, abstract, heading word, table of contents, key concepts, original title, tests & measures]

41. Suicide, Attempted/ or Suicide/ or Suicidal Ideation/

42. suicid*.mp. [mp=title, abstract, heading word, table of contents, key concepts, original title, tests & measures]

43. Substance-Related Disorders/

44. substance dependence.mp. [mp=title, abstract, heading word, table of contents, key concepts, original title, tests & measures]

45. substance abuse.mp. [mp=title, abstract, heading word, table of contents, key concepts, original title, tests & measures]

46. addiction.mp. [mp=title, abstract, heading word, table of contents, key concepts, original title, tests & measures]

47. Behavior, Addictive/

48. "substance use disorder".mp.

49. "drug use".mp. [mp=title, abstract, heading word, table of contents, key concepts, original title, tests & measures]

50. Personality Disorders/

51. personality disorder.mp. [mp=title, abstract, heading word, table of contents, key concepts, original title, tests & measures]

52. DEMENTIA, VASCULAR/ or DEMENTIA/ or FRONTOTEMPORAL DEMENTIA/ or DEMENTIA, MULTI-INFARCT/

53. dementia.mp. [mp=title, abstract, heading word, table of contents, key concepts, original title, tests & measures]

54. TOBACCO SMOKING/ or "TOBACCO USE DISORDER"/ or TOBACCO/ or "TOBACCO USE"/ or SMOKING, NON-TOBACCO PRODUCTS/

55. tobacco.mp. [mp=title, abstract, heading word, table of contents, key concepts, original title, tests & measures]

56. Tobacco Products/

57. cigarette.mp. [mp=title, abstract, heading word, table of contents, key concepts, original title, tests & measures]

58. PIPE SMOKING/ or COCAINE SMOKING/ or CIGARETTE SMOKING/ or SMOKING/ or CIGAR SMOKING/ or MARIJUANA SMOKING/

59. smoking.mp. [mp=title, abstract, heading word, table of contents, key concepts, original title, tests & measures]

60. COCAINE-RELATED DISORDERS/ or COCAINE/ or CRACK COCAINE/ or COCAINE SMOKING/

61. cocaine.mp. [mp=title, abstract, heading word, table of contents, key concepts, original title, tests & measures]

62. ALCOHOLS/

63. alcohol.mp. [mp=title, abstract, heading word, table of contents, key concepts, original title, tests & measures]

64. Opiate Alkaloids/

65. opiate.mp. [mp=title, abstract, heading word, table of contents, key concepts, original title, tests & measures]

66. HEROIN/ or HEROIN DEPENDENCE/

67. heroin.mp. [mp=title, abstract, heading word, table of contents, key concepts, original title, tests & measures]

68. AMPHETAMINE-RELATED DISORDERS/ or AMPHETAMINE/

69. amphetamine.mp. [mp=title, abstract, heading word, table of contents, key concepts, original title, tests & measures]

70. Mental Health/

71. mental health.mp. [mp=title, abstract, heading word, table of contents, key concepts, original title, tests & measures]

72. 3 or 4 or 5 or 6 or 7 or 8 or 9 or 10 or 11 or 12 or 13 or 14 or 15 or 16 or 17 or 18 or 19 or 20 or 21 or 22 or 23 or 24 or 25 or 26 or 27 or 28 or 29 or 30 or 31 or 32 or 33 or 34 or 35 or 36 or 37 or 38 or 39 or 40 or 41 or 42 or 43 or 44 or 45 or 46 or 47 or 48 or 49 or 50 or 51 or 52 or 53 or 54 or 55 or 56 or 57 or 58 or 59 or 60 or 61 or 62 or 63 or 64 or 65 or 66 or 67 or 68 or 69 or 70 or 71

73. 1 or 2

74. 72 and 73

75. limit 74 to humans
